# Synthesis and structure of 2-amino-1-methylbenzimid­azolium tetra­kis­[4,4,4-tri­fluoro-1-(1,3-thia­zol-2-yl)butane-1,3-dionato-κ^2^*O*,*O*′]cerium(III)

**DOI:** 10.1107/S2056989025010266

**Published:** 2025-11-18

**Authors:** Feruza Fayzullayeva, Sardor Murodov, Kambarali Turgunov, Mushtariybegim Sunnatillayeva, Bakhtigul Ruzieva, Bakhodir Tashkhodjaev, Shakhlo Daminova

**Affiliations:** a Uzbekistan–Japan Innovation Centre of Youth, University Street 2B, Tashkent 100095, Uzbekistan; b National University of Uzbekistan named after Mirzo Ulugbek University Street 4 Tashkent 100174 Uzbekistan; c Institute of the Chemistry of Plant Substances, Uzbekistan Academy of Sciences, Mirzo Ulugbek Str. 77, Tashkent 100170, Uzbekistan; d University Alfraganus, 100190, Tashkent, Uzbekistan; University of Aberdeen, United Kingdom

**Keywords:** crystal structure, cerium, Hirshfeld surface, multi-ligand complex system

## Abstract

The title compound, (C_8_H_10_N_3_)[Ce(C_7_H_3_F_3_NO_2_S)_4_], crystallizes with two cations and two anions in the asymmetric unit, with ion pairs linked by N—H⋯O hydrogen bonds. The Ce^3+^ cations adopt near regular square-anti­prismatic coordination geometries, arising from four *O*,*O*-bidentate ligands. Numerous N—H⋯F hydrogen bonds, as well as weak contacts such as C—H⋯F and C—H⋯N inter­actions occur in the extended structure. Hirshfeld surface analysis indicates that 34.8% of inter­molecular inter­actions are attributed to F⋯H/H⋯F, 13.2% to H⋯C/C⋯H, 11.1% to H⋯H, 6.6% to H⋯S/S⋯H and 5.5% to H⋯O/O⋯H contacts.

## Abstract 2

In the complex anion in the title compound, the cerium cation adopts a near regular square-anti­prismatic coordination geometry arising from four O,O-bidentate ligands.

## 1. Chemical context

Cerium(III) complexes are of inter­est due to their diverse coordination chemistry and potential applications in catalysis, luminescence, and materials science (Nehra *et al.*, 2022 ▸). β-Diketone ligands, such as 4,4,4-tri­fluoro-1-(1,3-thia­zol-2-yl)butane-1,3-dione, are well known for their strong chelating ability toward lanthanide ions, forming stable complexes with inter­esting structural features (Tubau *et al.*, 2024 ▸; Park *et al.*, 2022 ▸). Another widely studied ligand, 4,4,4-tri­fluoro-1-(2-thien­yl)butane-1,3-dione, commonly known as TTA, has been used in the synthesis of lanthanide complexes for the investigation of their intense fluorescent properties. The intra­molecular energy-transfer process from the 4,4,4-tri­fluoro-1-(2-thien­yl)butane-1,3-dionate (TTA) ligand to Eu^III^ ions in bis- and tris-(TTA) complexes was evaluated for the first time, and their photoluminescent and triboluminescent properties were also investigated (Teotonio *et al.*, 2008 ▸). The influence of incorporating various auxiliary compounds on the photoluminescent characteristics of Langmuir–Blodgett films of Eu(TTA)_3_Phen and Sm(TTA)_3_Phen complexes was investigated, showing a significant enhancement of luminescence and improved film ordering (Zhang *et al.*, 1997 ▸). In a subsequent study, new *Ln*^III^ complexes with the primary ligand 4,4,4-tri­fluoro-1-(2-thien­yl)butane-1,3-dione (TTA) and the auxiliary ligand *N*-methyl-ɛ-caprolactam were synthesized and structurally characterized, and their crystal structures and photoluminescent properties were investigated (Borges *et al.*, 2016 ▸). The use of lutetium porphyrinoid complexes as efficient triplet photosensitizers for fiber-optic upconversion *via* triplet–triplet annihilation (TTA) with high quantum yield and demonstrated suitability for live-cell bioimaging was also shown (Yang *et al.*, 2018 ▸). The photophysical behavior of Eu(TTA)_3_ complexes with new 1,10-phenanthroline derivatives was comprehensively studied by TD-DFT methods and experimental spectroscopy, revealing enhanced sensitization of the TTA ligand and several new nonradiative deactivation pathways (Silva *et al.*, 2024 ▸). As part of our studies in this area, we synthesized the complex (C_8_H_10_N_3_)^+^·[Ce(C_7_H_3_F_3_NO_2_S)_4_]^−^ (**I**) and we now describe its structure.
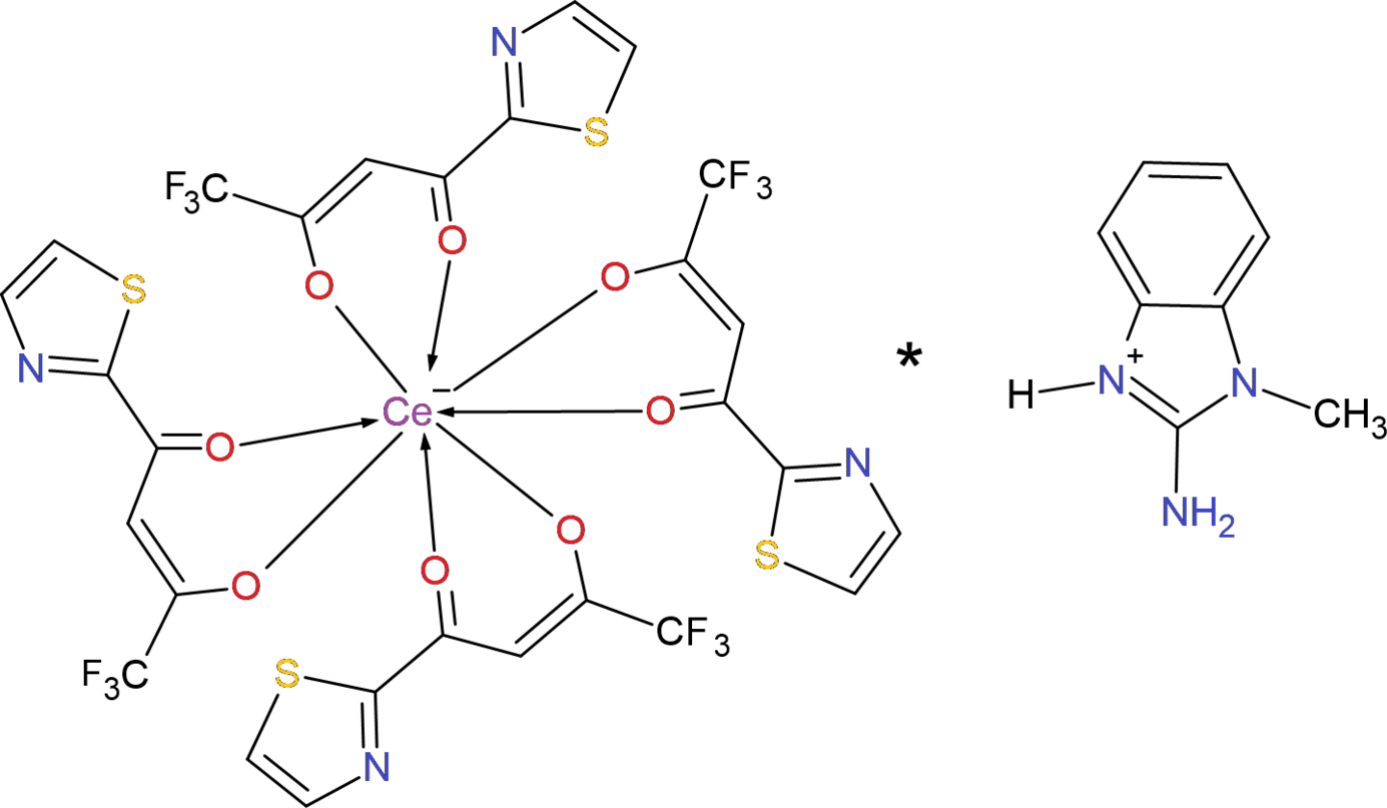


## 2. Structural commentary

**Figure 1:**
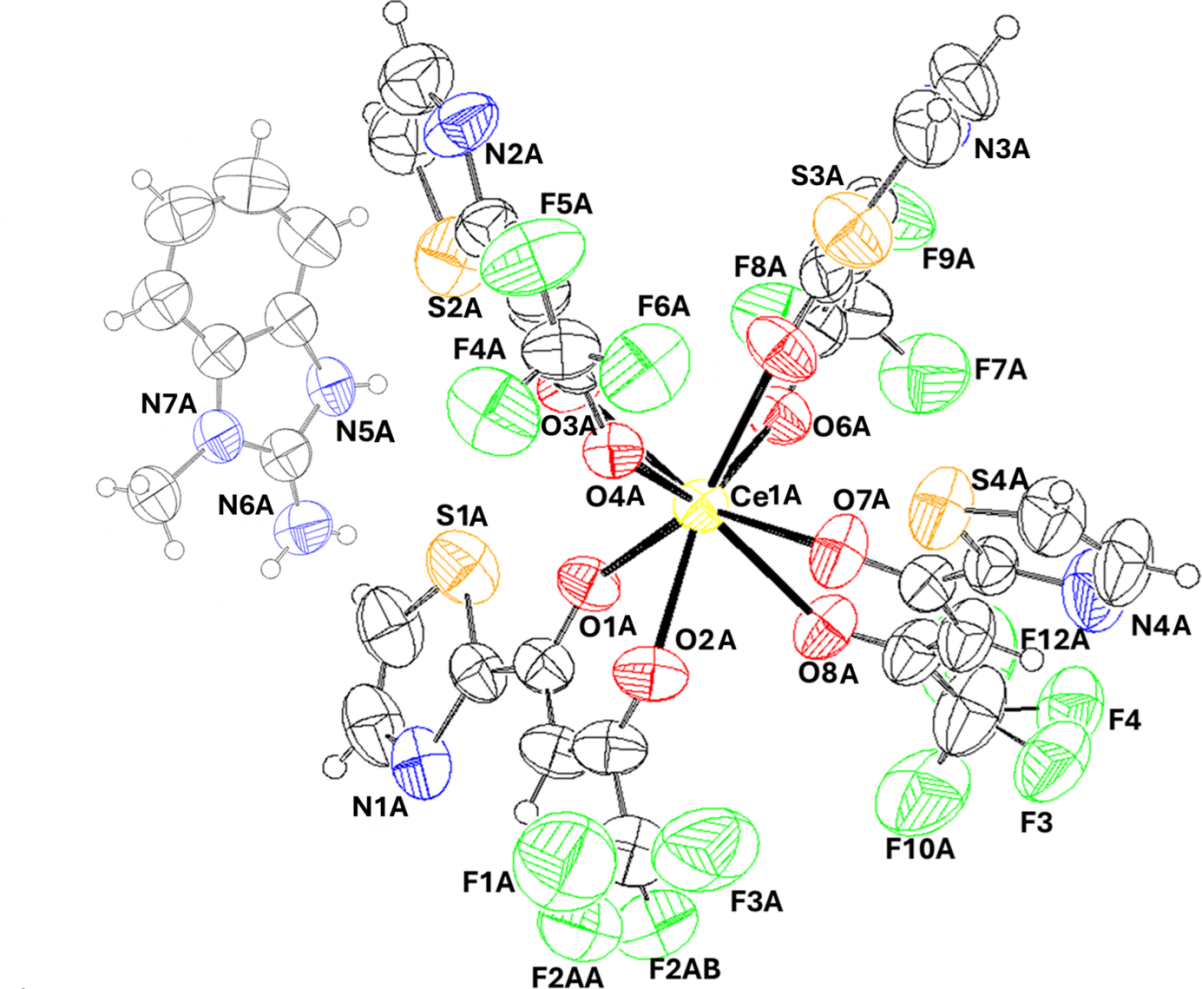
The mol­ecular structure of the Ce1*A* anion and N5*A* anion in (**I**), showing 50% probability ellipsoids.

Compound (**I**) (Fig. 1 ▸) crystallizes in the triclinic system with space group *P*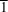. The asymmetric unit contains two crystallographically independent complex mol­ecules and two 2-amino-1-methyl­benzimidazole cations (*Z*′ = 2). Both Ce^3+^ ions are located at general positions and are coordinated by eight donor atoms from four *O*,*O*-bidentate 4,4,4-tri­fluoro-1-(1,3-thia­zol-2-yl)butane-1,3-dione (TFTB) ligands, forming a square-anti­prismatic geometry (Table 1 ▸). The inner coordination sphere of the complex carries a formal charge of −1; thus, the second ligand, 2-amino-1-methyl­benzimidazole (MAB), bearing a protonated nitro­gen atom and a charge of +1, is situated in the outer coordination sphere, ensuring charge balance and contributing to supra­molecular inter­actions. The Ce—O bond lengths range from 2.405 (3) to 2.580 (3) Å for Ce1*A* and from 2.434 (3) to 2.543 (3) Å for Ce1*B* (Table 1 ▸). These values fall within the typical range for Ce^3+^—O bonds and indicate a slightly distorted coordination geometry around the metal center (Estevenon *et al.*, 2023 ▸). The TFTB ligand forms a six-membered chelate ring, and the O—Ce—O bite angles, ranging from 68.84 (12)–70.26 (11)° for Ce1*A* and 68.95 (12)–70.40 (12)° for Ce1*B*, suggest a mildly strained chelating geometry.

## 3. Supra­molecular features

The packing of compound (**I**) is largely determined by directional hydrogen bonds of the N—H⋯O and N—H⋯F types, as well as by a multitude of weak C—H⋯F and C—H⋯N contacts. These inter­actions perform different but complementary functions: the MAB cations act as local N—H donors, which form the strongest and most directional contacts with the acetonyl O atoms of the TFTB ligand and with the most electronegative F atoms. As a result, the N—H⋯O and N—H⋯F bonds directly ‘attach’ the MAB cations to the periphery of the TFTB complexes and serve as ‘nodal’ points of the supra­molecular network. The complete list of inter­molecular hydrogen bonds and geometric parameters are given in Table 2 ▸.

**Figure 2:**
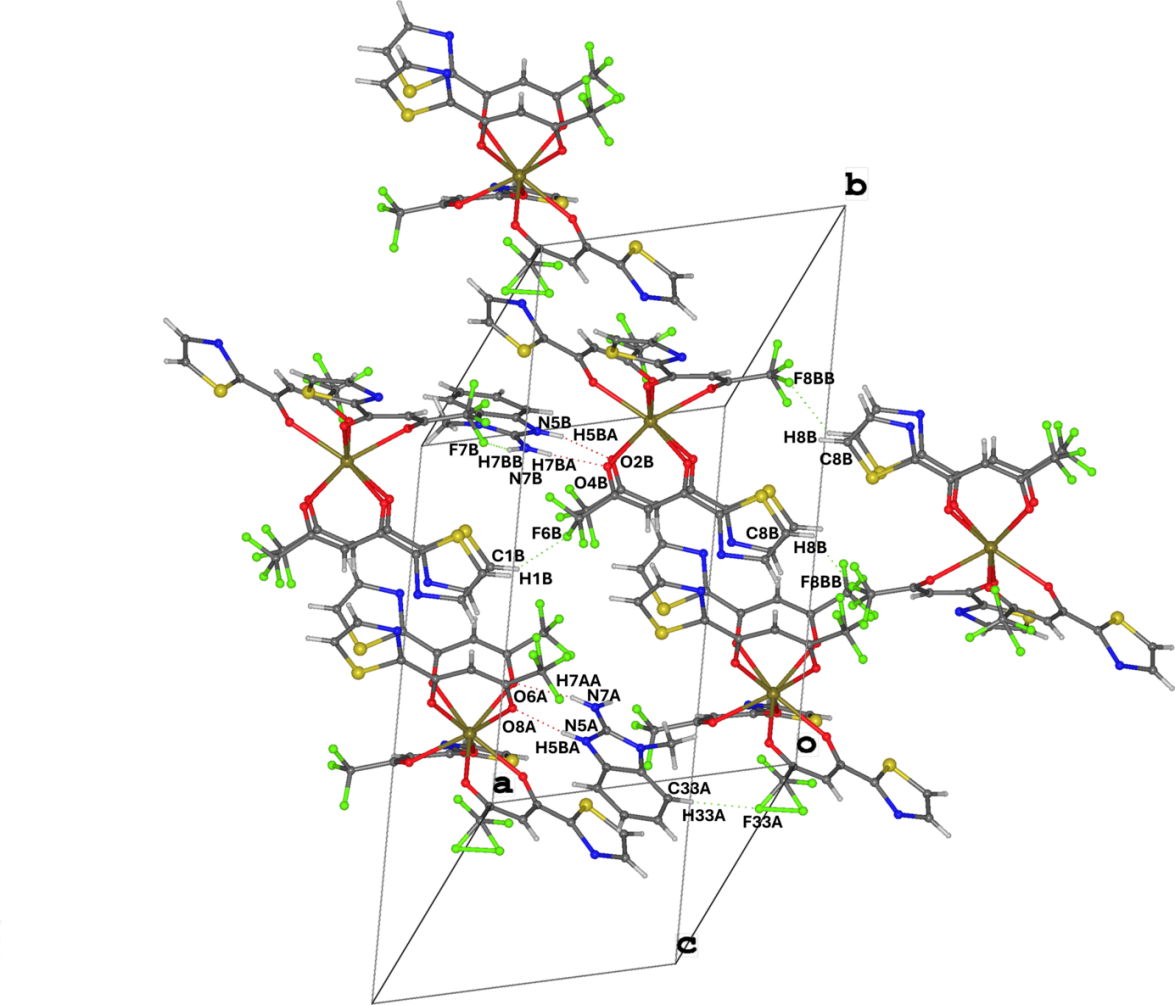
Packing diagram of (**I**) showing the N—H⋯O and N—H⋯F hydrogen bonds and C—H⋯F and C—H⋯N weak contacts resulting in chains propagating along [001].

Weak C—H⋯F and C—H⋯N contacts are widely distributed throughout the structure and consolidate the packing between adjacent complexes. In some cases, C—H donors are methyl or aromatic C—H groups of TFTB and MAB, and acceptors are fluorinated substituents or nitro­gen/oxygen atoms. Such contacts often appear as ‘bridges’ between chains, linking primary N—H networks into layered fragments. As a result, a multi-level supra­molecular architecture is formed: directional N—H bonds form the main extended axis of the network, weak C—H⋯*X* contacts compact the packing and provide cross-linking between chains, and secondary contacts fix the relative orientation of adjacent mol­ecules. Topologically, it is observed that N—H⋯O/N—H⋯F bonds create linear or slightly branched chains extending along the [001] axis and forming the primary supra­molecular subnetwork, C—H⋯F contacts serve as ‘seams’ between chains, often realizing a regular repetition of contact nodes and setting the periodicity of packing in the plane (Fig. 2 ▸).

**Figure 3:**
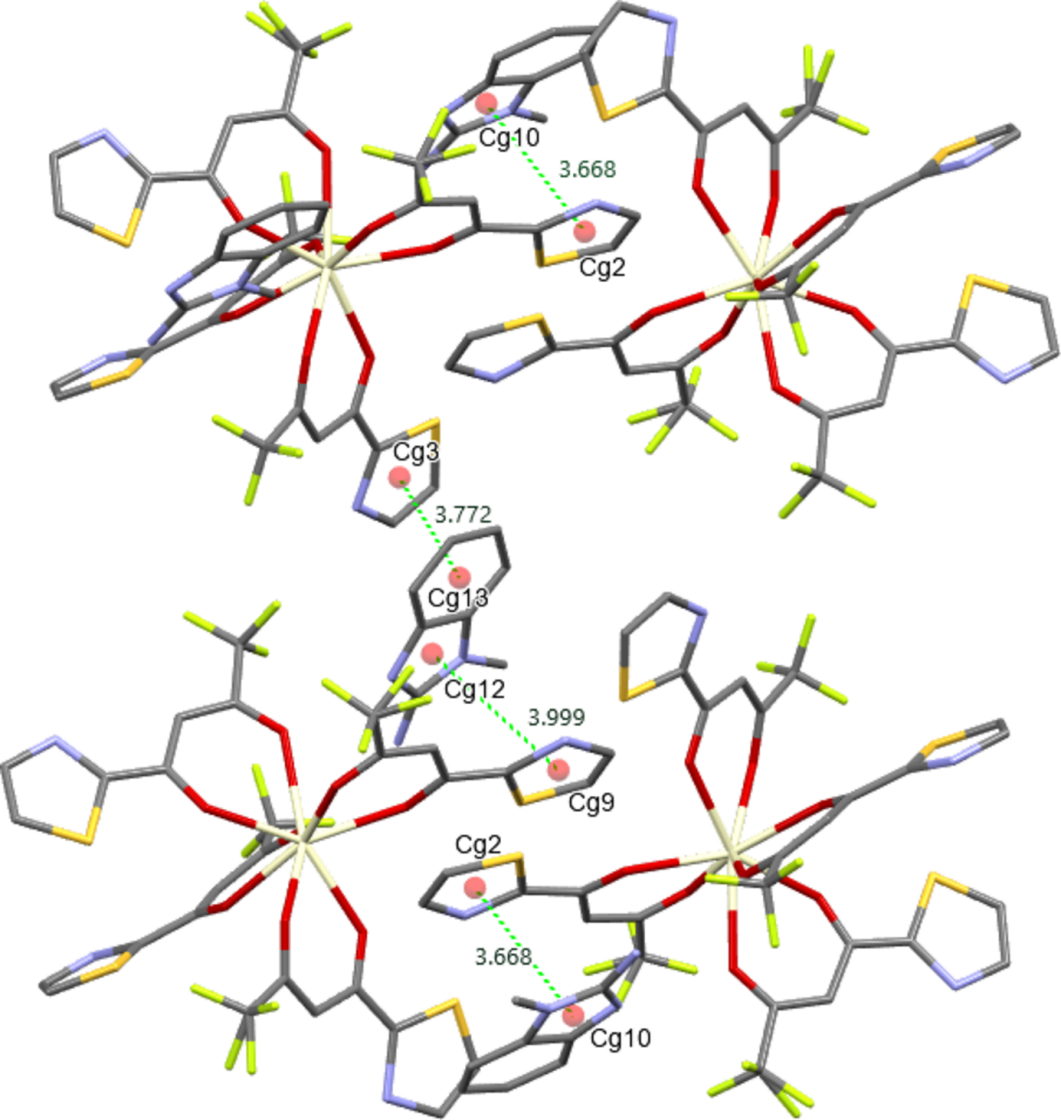
The π–π contacts in the structure of (**I**).

In addition, (**I**) exhibits π-stacking inter­actions that enhance the packing perpendicular to the direction of the hydrogen-bonded chains. Classical aromatic π–π stacking contacts are observed between the following pairs of rings: *Cg*2⋯*Cg*10, *Cg*3⋯*Cg*13, *Cg*9⋯*Cg*12, and *Cg*10⋯*Cg*2 (Table 2 ▸). These *π–π* stacks form columns of aromatic moieties along which additional consolidation occurs through the overlap of π-clouds; the columns, in turn, are linked to hydrogen-bonded chains to form a three-dimensional network. Additional contacts involving π-systems include *X*—H⋯*Cg* inter­actions. Such contacts can be viewed as weak hydrogen/electrostatic bonds, where donor moieties (*X*—H or electron-negative *Y*—*X* groups) inter­act with the π-surface, facilitating the orientation of aromatic systems and reducing the freedom of rotation of peripheral moieties (Fig. 3 ▸). The geometric parameters of all mentioned π-inter­actions are given in Table 2 ▸ along with centroid definitions.

It is important to note the influence of dispersion interactions and positional disorder of the fluorine substituents on the crystal packing. The outer coordination sphere contains four disorder zones (*A*–*D*; see *Refinement* section), where alternative positions of -atoms are possible. Partial occupancy and statistical distortion of the fluorine atoms result in some contacts (especially C—H⋯F and F⋯π) existing in alternative packing arrangements and statistically averaged over the crystal. This means that in the real structure a family of configurations of close energy is observed, where some contacts are retained in one copy of the cell, while alternative connections are realized in the other. From a practical point of view, such multi-position occupancy increases the number of possible weak contacts and gives a ‘denser’ statistical picture of the packing than follows from consideration of only one fixed configuration.

## 4. Hirshfeld surface and void analysis

**Figure 4:**
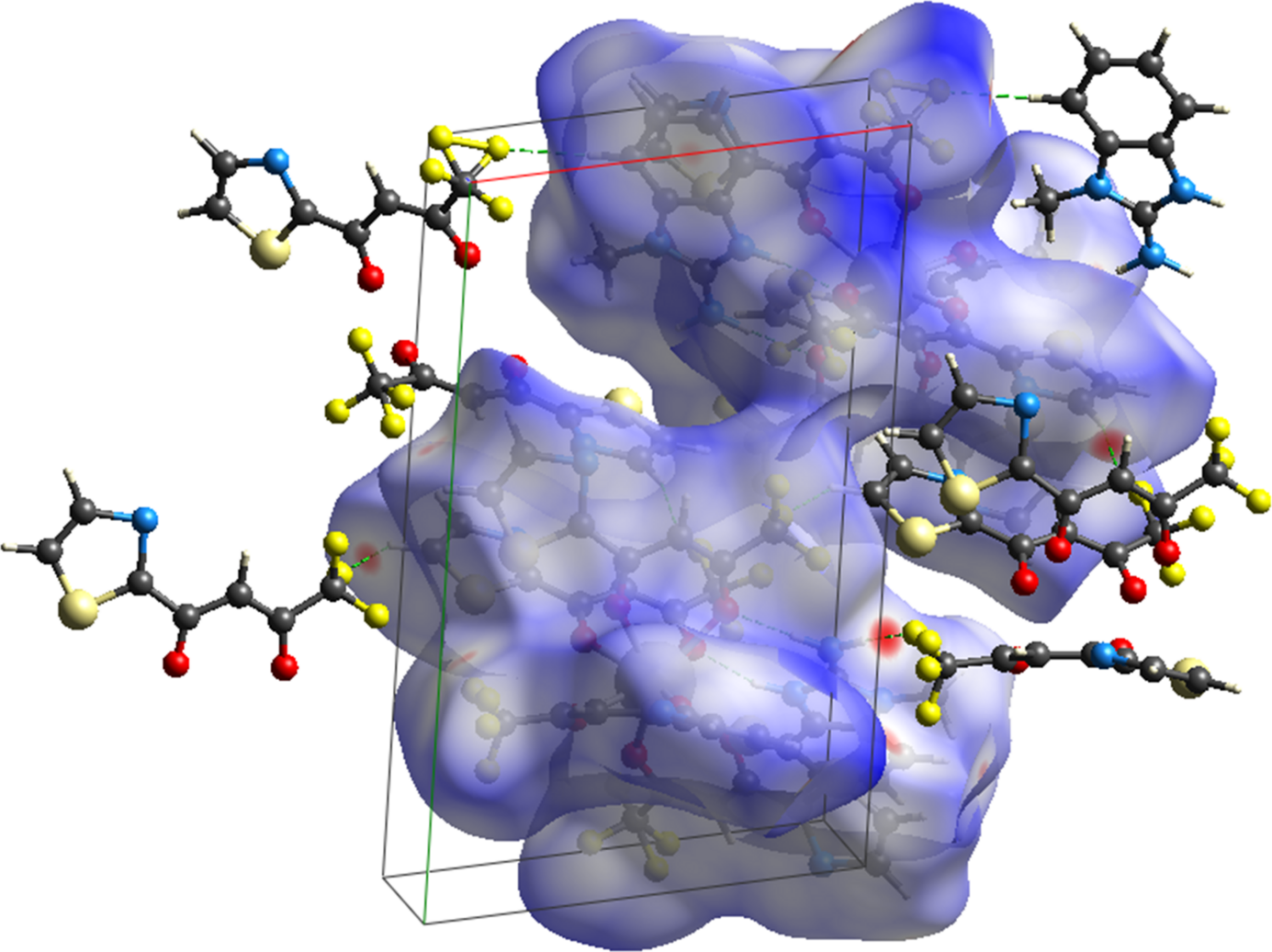
Hirshfeld surface of (**I**) mapped over *d*_norm_ showing close inter­molecular contacts (red spots).

**Figure 5:**
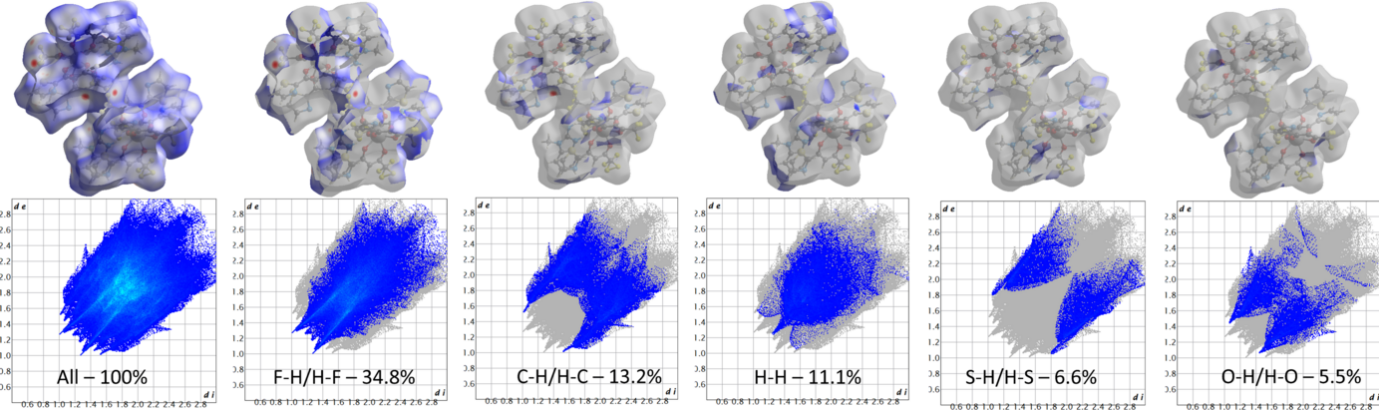
*(a*) The full two-dimensional fingerprint plots for (**I**), showing all inter­actions and (*b*)–(*f*) those delineated into specified inter­actions.

The Hirshfeld surface for (**I**) was calculated using *CrystalExplorer 21.5* (Spackman *et al.*, 2021 ▸). The red spots on the *d*_norm_ map (Fig. 4 ▸) identify the most prominent close contacts in the structure and therefore highlight the locations of strong inter­molecular inter­actions (notably N—H⋯O, N—H⋯F and short C—H⋯F contacts), whereas the blue areas correspond to regions where inter­molecular distances exceed the expected van der Waals separations. The overall two-dimensional fingerprint plot is shown in Fig. 5 ▸*a* and provides a visual summary of all pairwise contacts; characteristic features and spikes in the fingerprint plots can be related to specific inter­action types (see below).

The greatest contribution to the Hirshfeld surface arises from F⋯H/H⋯F contacts (Fig. 5 ▸*b*), accounting for 34.8%. This dominant fraction reflects the high fluorine content of the TFTB ligands combined with multiple hydrogen donors (N—H and C—H), and is consistent with the intense red spots observed near F atoms on the *d*_norm_ map. Contributions from H⋯C/C⋯H (Fig. 5 ▸*c*), H⋯H (Fig. 5 ▸*d*), H⋯S/S⋯H (Fig. 5 ▸*e*), and H⋯O/O⋯H (Fig. 5 ▸*f*) contacts are 13.2%, 11.1%, 6.6%, and 5.5%, respectively. The H⋯C component largely arises from C—H⋯π-type inter­actions and close contacts involving aromatic rings and aliphatic C—H groups, the H⋯H contribution reflects pervasive van der Waals packing between non-polar fragments, while the H⋯S and H⋯O fractions signal the participation of thia­zole S atoms and carbonyl O atoms in secondary hydrogen-bonding and electrostatic contacts.

The less significant remaining contributions include F⋯F, N⋯C/C⋯N, F⋯S/S⋯F, N⋯H/H⋯N, C⋯C, F⋯C/C⋯F, N⋯F/F⋯N, N⋯O/O⋯N, N⋯N, N⋯S/S⋯N, S⋯C/C⋯S, and C⋯O/O⋯C inter­actions, contributing 5.3%, 5.3%, 4%, 3.9%, 2.6%, 2%, 2%, 1.5%, 1%, 0.8%, 0.3% and 0.3%, respectively. Collectively, these minor fractions reflect a rich variety of weak electrostatic and dispersive contacts and also capture effects of the observed fluorine disorder: many of the smaller contributions (for example F⋯F, F⋯C and F⋯S) arise from alternative F-atom positions and partial occupancies, which broaden the distribution of contact types on the fingerprint plots. Altogether, the Hirshfeld analysis complements the supra­molecular description given above and qu­anti­fies the relative importance of F-mediated inter­actions, C—H and π-contacts, and heteroatom-involving contacts in defining the crystal packing.

**Figure 6:**
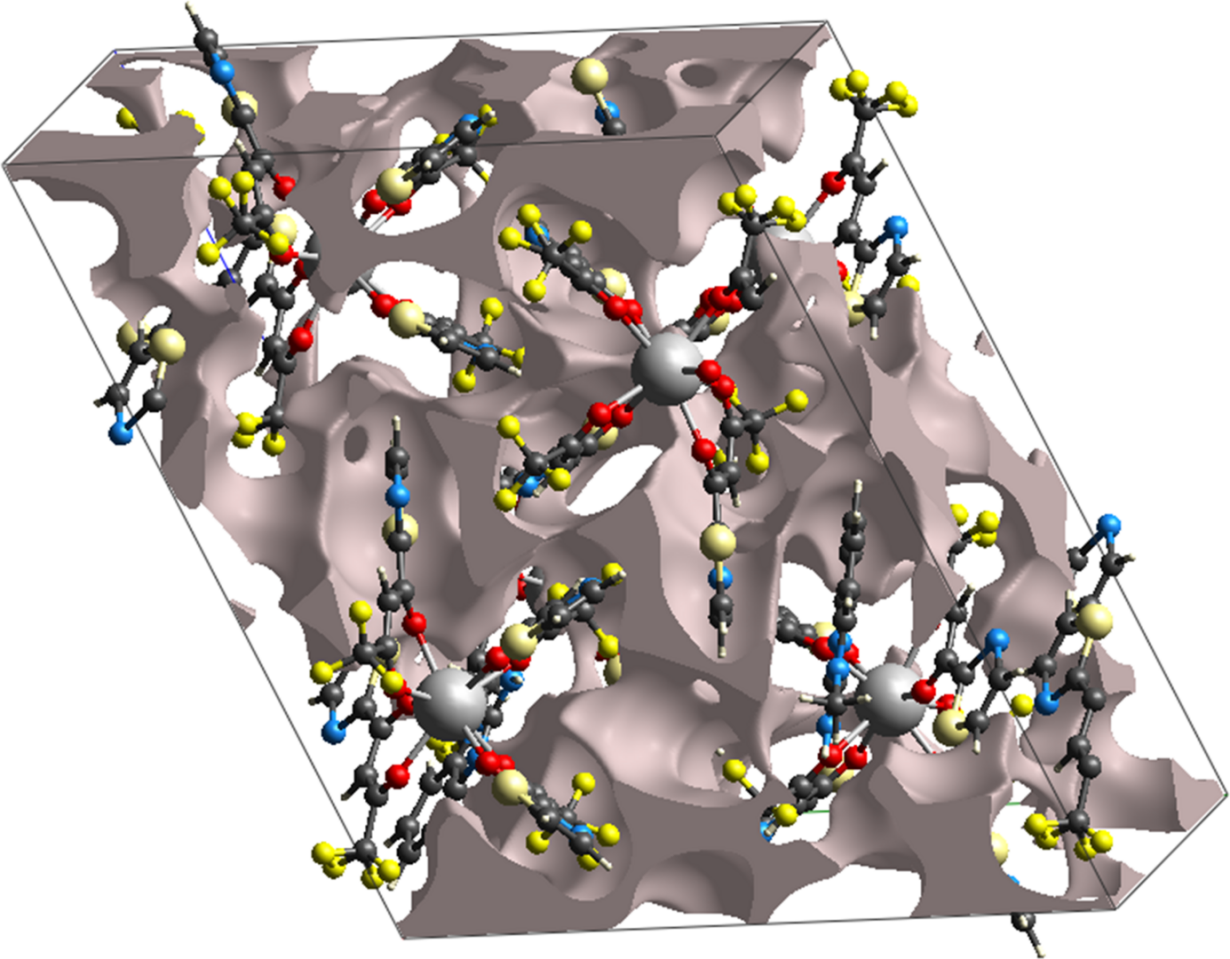
The void surface packing of (**I**).

A void analysis was performed using the pro-crystal electron density approach (Turner *et al.*, 2011 ▸). The void surface was defined as an isosurface of the pro-crystal density and computed for the entire unit cell, where the isosurface inter­sects the unit-cell boundaries to define closed volumes. The total void volume in the unit cell is 907 Å^3^, corresponding to 19% of the unit-cell volume. For *Z* = 4 this corresponds to a void volume per formula unit of ∼227 Å^3^ We stress that absolute void volumes depend on calculation settings (in particular the probe radius and isovalue used to define the isosurface); the value reported here was obtained with the standard parameters of the applied procedure. Void analysis characterises the amount and topology of unoccupied space (isolated cavities *versus* inter­connected channels) and is therefore useful for comparing packing efficiency between related structures and for identifying potential solvent-accessible regions. The void surface is shown in (Fig. 6 ▸).

## 5. Database survey

A search of the Cambridge Structural Database (CSD version 2024.2.0; Groom *et al.*, 2016 ▸) revealed three structurally related compounds containing a fragment similar to that of compound (**I**). Notably, no analogous structures containing the TFTB ligand were found in the database. In the case of the MAB ligand, 73 related structures were identified, including those with the following CSD refcodes: BOVMAB (Kadirova *et al.*, 2009 ▸), FUFWIM (Antsyshkina *et al.*, 1987 ▸), GOKTOP (Garnovskii *et al.*, 1998 ▸) and JABTOY (Garnovskii *et al.*, 2015 ▸).

## 6. Synthesis and crystallization

**Figure 7:**
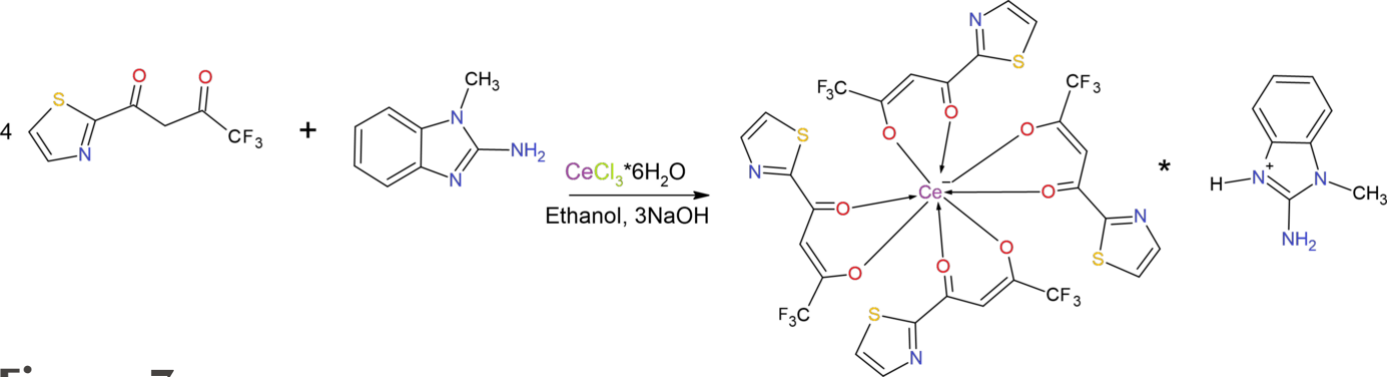
Synthesis scheme for (**I**).

TFTB (3.0 mmol) was dissolved in ethanol and stirred with an alcoholic solution of an alkali (3.0 mmol) for 1 h. After that, an ethanol solution of CeCl_3_·6H_2_O salt (1.0 mmol) was added dropwise to the reaction mixture (Fig. 7 ▸). The resulting mixture was stirred continuously at room temperature for 4 h. Subsequently, an alcoholic solution of MAB (1.0 mmol) was added dropwise to the stirred mixture, and stirring was continued for an additional 2 h. The precipitate was filtered, washed several times with ethanol, and dried in air. Since the resulting material is readily soluble in di­methyl­formamide (DMF), it was recrystallized from this solvent to obtain well-formed dark-yellow single crystals suitable for structural and further physicochemical studies. The synthesis scheme is shown in Fig. 7 ▸.

## 7. Refinement

Crystal data, data collection and structure refinement details are summarized in Table 3 ▸. C–bound hydrogen atoms were placed geometrically and treated as riding atoms, with C—H = 0.93–0.97 Å. *U*_iso_(H) was set to 1.5*U_eq_*(C) for methyl hydrogen atoms and 1.2*U*_eq_(C) otherwise. Several fluorine atoms were found to be disordered and were modelled as follows: atoms F8*BA* and F8*BB* occupy adjacent sites with fixed occupancies of 0.50 each, indicating a two-position statistical disorder. Atoms F1 and F2 were refined using a free variable card, with refined occupancies of 0.63 (3) for F1 and 0.37 (3) for F2, indicating a preferred occupancy for F1. Atoms F2*AA* and F2*AB* were modelled using an FVAR card with refined occupancies of 0.628 (17) and 0.372 (17), respectively. Atoms F3 and F4 were modelled with fixed occupancies of 0.50 each.

## Supplementary Material

Crystal structure: contains datablock(s) global, I. DOI: 10.1107/S2056989025010266/hb8162sup1.cif

Structure factors: contains datablock(s) I. DOI: 10.1107/S2056989025010266/hb8162Isup2.hkl

CCDC reference: 2502995

Additional supporting information:  crystallographic information; 3D view; checkCIF report

## Figures and Tables

**Table 1:** Selected geometric parameters (Å, °)

Ce1*A*—O2*A*	2.405 (3)	Ce1*B*—O7*B*	2.434 (3)
Ce1*A*—O7*A*	2.436 (3)	Ce1*B*—O3*B*	2.444 (4)
Ce1*A*—O5*A*	2.443 (4)	Ce1*B*—O6*B*	2.453 (3)
Ce1*A*—O3*A*	2.443 (3)	Ce1*B*—O1*B*	2.453 (4)
Ce1*A*—O1*A*	2.457 (4)	Ce1*B*—O8*B*	2.460 (4)
Ce1*A*—O4*A*	2.467 (3)	Ce1*B*—O5*B*	2.476 (4)
Ce1*A*—O6*A*	2.516 (4)	Ce1*B*—O4*B*	2.524 (4)
Ce1*A*—O8*A*	2.580 (3)	Ce1*B*—O2*B*	2.543 (3)
			
*Cg*2⋯*Cg*10	3.668 (4)	*Cg*9⋯*Cg*12	3.999 (4)
*Cg*3⋯*Cg*13^i^	3.772 (5)	*Cg*10⋯*Cg*2	3.668 (4)
			
O1*A*—Ce1*A*—O2*A*	70.26 (11)	O7*B*—Ce1*B*—O8*B*	70.40 (12)
O3*A*—Ce1*A*—O4*A*	69.57 (11)	O5*B*—Ce1*B*—O6*B*	69.86 (12)
O5*A*—Ce1*A*—O6*A*	68.84 (12)	O3*B*—Ce1*B*—O4*B*	68.95 (12)
O7*A*—Ce1*A*—O8*A*	68.99 (11)	O1*B*—Ce1*B*—O2*B*	69.23 (11)

Symmetry code: (i) 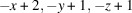.

**Table 2:** Hydrogen-bond geometry (Å, °) The rind centroids, some of which are referred to in the text and not the table, are defined as follows: *Cg*2 = S1*A*/C1*A*/C2*A*/N1*A*/C3*A*; *Cg*3 = S2*A*/C8*A*/C9*A*/N2*A*/C10*A*; *Cg*4 = S3*A*/C15*A*/C16*A*/N3*A*/C17*A*; *Cg*6 = S1*B*/C1*B*/C2*B*/N1*B*/C3*B*; *Cg*7 = S2*B*/C8*B*/C9*B*/N2*B*/C10*B*; *Cg*8 = S3*B*/C15*B*/C16*B*/N3*B*/C17*B*; *Cg*9 = S4*B*/C22*B*/C23*B*/N4*B*/C24*B*; *Cg*10 = N5*A*/C29*A*/C34*A*/N6*A*/C35*A*; *Cg*12 = N5*B*/C29*B*/C34*B*/N6*B*/C35*B*; *Cg*13 = C29*B*/C30*B*/C31*B*/C32*B*/C33*B*/C34*B*.

*D*—H⋯*A*	*D*—H	H⋯*A*	*D*⋯*A*	*D*—H⋯*A*
N5*B*—H5*BA*⋯O2*B*	0.86	1.98	2.817 (5)	166
N5*A*—H5*AA*⋯O8*A*	0.86	2.00	2.843 (5)	168
N7*B*—H7*BA*⋯O4*B*	0.86	2.21	3.055 (6)	169
N7*B*—H7*BB*⋯F7*B*^ii^	0.86	2.41	3.227 (7)	158
N7*A*—H7*AA*⋯O6*A*	0.86	2.23	3.051 (6)	160
N7*A*—H7*AB*⋯F6*A*^iii^	0.86	2.60	3.397 (7)	155
C33*B*—H33*B*⋯F10*B*^ii^	0.93	2.60	3.495 (9)	161
C1*B*—H1*B*⋯F6*B*^iii^	0.93	2.46	3.178 (8)	134
C33*A*—H33*A*⋯F3*A*^iii^	0.93	2.51	3.429 (11)	168
C9*A*—H9*A*⋯S1*B*^iv^	0.93	3.01	3.941 (8)	174
C2*A*—H2*A*⋯O6*B*^v^	0.93	2.63	3.433 (7)	145
C8*B*—H8*B*⋯F8*BB*^vi^	0.93	2.52	3.139 (14)	124
C9*A*—H9*A*⋯*Cg*6^iv^	0.93	2.85	3.656 (10)	145
C31*A*—H31*A*⋯*Cg*8^v^	0.93	2.91	3.612 (10)	133
C14*B*—F4*B*⋯*Cg*7^vii^	1.32 (1)	3.59 (1)	3.906 (8)	94 (1)
C14*B*—F5*B*⋯*Cg*7^vii^	1.33 (1)	3.51 (1)	3.906 (8)	97 (1)
C21*A*—F8*A*⋯*Cg*4^i^	1.34 (1)	3.45 (1)	4.119 (7)	111 (1)
*Cg*2⋯*Cg*10			3.668 (4)	
*Cg*3⋯*Cg*13^i^			3.772 (5)	
*Cg*9⋯*Cg*12			3.999 (4)	
*Cg*10⋯*Cg*2			3.668 (4)	

Symmetry codes: (i) 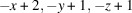; (ii) 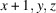; (iii) 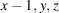; (iv) 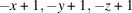; (v) 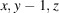; (vi) 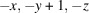; (vii) 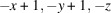.

**Table 3:** Experimental details

Crystal data	
Chemical formula	(C_8_H_10_N_3_)[Ce(C_7_H_3_F_3_NO_2_S)_4_]
*M*_r_	1176.96
Crystal system, space group	Triclinic, *P* 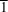
Temperature (K)	293
*a*, *b*, *c* (Å)	11.0242 (10), 19.7469 (17), 24.154 (2)
α, β, γ (°)	113.801 (4), 90.657 (4), 100.739 (4)
*V* (Å^3^)	4704.8 (7)
*Z*	4
Radiation type	Mo *K*α
μ (mm^−1^)	1.25
Crystal size (mm)	0.20 × 0.18 × 0.10

Data collection	
Diffractometer	Bruker APEXII CCD
Absorption correction	Multi-scan (*SADABS*; Krause *et al.*, 2015 ▸)
*T*_min_, *T*_max_	0.616, 0.746
No. of measured, independent and observed [*I* > 2σ(*I*)] reflections	243954, 21637, 15811
*R*_int_	0.098
(sin θ/λ)_max_ (Å^−1^)	0.651

Refinement	
*R*[*F*^2^ > 2σ(*F*^2^)], *wR*(*F*^2^), *S*	0.054, 0.166, 1.02
No. of reflections	21637
No. of parameters	1266
No. of restraints	210
H-atom treatment	H-atom parameters constrained
Δρ_max_, Δρ_min_ (e Å^−3^)	1.55, −1.28

Computer programs: *APEX3* and *SAINT* (Bruker, 2016 ▸), *SHELXT* (Sheldrick, 2015*a* ▸), *SHELXL* (Sheldrick, 2015*b* ▸) and *OLEX2* (Dolomanov *et al.*, 2009 ▸).

## supplementary crystallographic information

### 2-Amino-1-methylbenzimidazolium tetrakis[4,4,4-trifluoro-1-(1,3-thiazol-2-yl)butane-1,3-dionato-κ2O,O']cerium(III) Crystal data

**Table:**

C_8_H_10_N_3_^+^·C_28_H_12_CeF_12_N_4_O_8_S_4_^−^	*Z* = 4
*M**_r_* = 1176.96	*F*(000) = 2324
Triclinic, *P*1	*D*_x_ = 1.662 Mg m^−^^3^
*a* = 11.0242 (10) Å	Mo *K*α radiation, λ = 0.71073 Å
*b* = 19.7469 (17) Å	Cell parameters from 9791 reflections
*c* = 24.154 (2) Å	θ = 2.2–21.1°
α = 113.801 (4)°	µ = 1.25 mm^−^^1^
β = 90.657 (4)°	*T* = 293 K
γ = 100.739 (4)°	Block, colourless
*V* = 4704.8 (7) Å^3^	0.20 × 0.18 × 0.10 mm

### 2-Amino-1-methylbenzimidazolium tetrakis[4,4,4-trifluoro-1-(1,3-thiazol-2-yl)butane-1,3-dionato-κ2O,O']cerium(III) Data collection

**Table:**

Bruker APEXII CCD diffractometer	15811 reflections with *I* > 2σ(*I*)
φ and ω scans	*R*_int_ = 0.098
Absorption correction: multi-scan (SADABS; Krause *et al.*, 2015)	θ_max_ = 27.6°, θ_min_ = 1.8°
*T*_min_ = 0.616, *T*_max_ = 0.746	*h* = −14→14
243954 measured reflections	*k* = −25→25
21637 independent reflections	*l* = −31→31

### 2-Amino-1-methylbenzimidazolium tetrakis[4,4,4-trifluoro-1-(1,3-thiazol-2-yl)butane-1,3-dionato-κ2O,O']cerium(III) Refinement

**Table:**

Refinement on *F*^2^	Hydrogen site location: inferred from neighbouring sites
Least-squares matrix: full	H-atom parameters constrained
*R*[*F*^2^ > 2σ(*F*^2^)] = 0.054	*w* = 1/[σ^2^(*F*_o_^2^) + (0.084*P*)^2^ + 5.2141*P*] where *P* = (*F*_o_^2^ + 2*F*_c_^2^)/3
*wR*(*F*^2^) = 0.166	(Δ/σ)_max_ = 0.008
*S* = 1.02	Δρ_max_ = 1.55 e Å^−^^3^
21637 reflections	Δρ_min_ = −1.28 e Å^−^^3^
1266 parameters	Extinction correction: SHELXL-2019/2 (Sheldrick 2015b), Fc^*^=kFc[1+0.001xFc^2^λ^3^/sin(2θ)]^-1/4^
210 restraints	Extinction coefficient: 0.00073 (17)
Primary atom site location: dual	

### 2-Amino-1-methylbenzimidazolium tetrakis[4,4,4-trifluoro-1-(1,3-thiazol-2-yl)butane-1,3-dionato-κ2O,O']cerium(III) Special details

**Table:**

Geometry. All esds (except the esd in the dihedral angle between two l.s. planes) are estimated using the full covariance matrix. The cell esds are taken into account individually in the estimation of esds in distances, angles and torsion angles; correlations between esds in cell parameters are only used when they are defined by crystal symmetry. An approximate (isotropic) treatment of cell esds is used for estimating esds involving l.s. planes.

### 2-Amino-1-methylbenzimidazolium tetrakis[4,4,4-trifluoro-1-(1,3-thiazol-2-yl)butane-1,3-dionato-κ2O,O']cerium(III) Fractional atomic coordinates and isotropic or equivalent isotropic displacement parameters (Å^2^)

**Table:**

	*x*	*y*	*z*	*U*_iso_*/*U*_eq_	Occ. (<1)
Ce1A	0.99578 (2)	0.22987 (2)	0.29281 (2)	0.04908 (9)	
Ce1B	0.51457 (2)	0.72546 (2)	0.20896 (2)	0.05334 (10)	
S1B	0.09249 (14)	0.57694 (11)	0.22978 (9)	0.0817 (5)	
S4A	1.40308 (15)	0.34963 (12)	0.24180 (9)	0.0906 (5)	
S3A	1.34387 (16)	0.45117 (11)	0.41721 (10)	0.0960 (5)	
S1A	0.58212 (16)	0.07756 (10)	0.30717 (9)	0.0894 (5)	
S2A	0.78110 (19)	0.23447 (13)	0.48483 (10)	0.1026 (6)	
S2B	0.1703 (2)	0.51639 (14)	0.07058 (11)	0.1209 (8)	
S3B	0.7203 (2)	0.80383 (14)	0.04237 (11)	0.1147 (7)	
N4B	0.9347 (3)	1.01269 (15)	0.33724 (14)	0.0351 (7)	
S4B	0.9491 (3)	0.89070 (17)	0.23137 (17)	0.1476 (10)	
O4A	1.1728 (3)	0.20883 (19)	0.34269 (15)	0.0559 (8)	
O2B	0.6030 (3)	0.6916 (2)	0.28983 (17)	0.0637 (9)	
O6A	0.8549 (3)	0.3228 (2)	0.32702 (16)	0.0631 (9)	
O4B	0.6603 (3)	0.6375 (2)	0.16208 (17)	0.0653 (9)	
O7B	0.7053 (3)	0.82174 (19)	0.24455 (16)	0.0610 (8)	
O3A	0.9443 (3)	0.2266 (2)	0.38991 (16)	0.0647 (9)	
O6B	0.3338 (3)	0.7580 (2)	0.17319 (16)	0.0590 (8)	
O2A	0.8131 (3)	0.13206 (18)	0.27198 (16)	0.0590 (8)	
O8B	0.4871 (3)	0.8290 (2)	0.30468 (17)	0.0674 (9)	
O1A	1.0240 (3)	0.1132 (2)	0.20866 (17)	0.0694 (10)	
O7A	1.1506 (3)	0.2768 (2)	0.23857 (18)	0.0731 (10)	
O8A	0.8935 (3)	0.2343 (2)	0.19803 (17)	0.0662 (9)	
O5B	0.5639 (3)	0.7609 (2)	0.12319 (17)	0.0663 (9)	
F6A	1.4157 (4)	0.2650 (3)	0.3833 (2)	0.1197 (15)	
N2B	0.3043 (4)	0.4024 (2)	0.01765 (19)	0.0652 (12)	
O1B	0.3489 (3)	0.6589 (2)	0.2469 (2)	0.0742 (11)	
N6B	1.0456 (4)	0.7995 (3)	0.3244 (2)	0.0676 (11)	
F5A	1.3851 (4)	0.2645 (3)	0.4692 (2)	0.1293 (18)	
F1B	0.7520 (4)	0.6077 (3)	0.3126 (2)	0.1155 (15)	
F8A	0.7584 (4)	0.4900 (2)	0.4165 (2)	0.1108 (14)	
F4B	0.8593 (4)	0.5810 (3)	0.1170 (2)	0.1273 (17)	
O3B	0.4078 (3)	0.6110 (2)	0.1229 (2)	0.0773 (11)	
F3B	0.7005 (4)	0.6840 (3)	0.3954 (2)	0.1145 (14)	
O5A	1.1085 (3)	0.3544 (2)	0.3645 (2)	0.0746 (11)	
F7A	0.7229 (5)	0.4255 (3)	0.3198 (2)	0.1289 (17)	
F5B	0.7505 (4)	0.4719 (3)	0.0667 (2)	0.1245 (17)	
F9A	0.6541 (4)	0.3768 (3)	0.3790 (2)	0.1198 (16)	
N5B	0.8473 (4)	0.7753 (3)	0.3316 (2)	0.0671 (11)	
H5BA	0.769088	0.757069	0.321953	0.081*	
F6B	0.7732 (5)	0.5210 (3)	0.1642 (3)	0.1377 (18)	
F2B	0.6130 (4)	0.5662 (3)	0.3582 (3)	0.1258 (17)	
F4A	1.3596 (4)	0.1619 (3)	0.3902 (3)	0.1299 (17)	
N6A	0.4548 (4)	0.1307 (3)	0.1971 (2)	0.0714 (12)	
N5A	0.6462 (4)	0.1489 (3)	0.1750 (2)	0.0690 (12)	
H5AA	0.722801	0.169851	0.177105	0.083*	
C13A	1.2049 (4)	0.2321 (3)	0.3980 (2)	0.0529 (10)	
F8BB	0.1182 (13)	0.7096 (7)	0.0510 (7)	0.103 (4)	0.5
N3B	0.5116 (5)	0.7526 (3)	−0.0342 (2)	0.0781 (14)	
N1A	0.5730 (4)	−0.0486 (3)	0.2055 (2)	0.0691 (12)	
F7B	0.0929 (4)	0.6996 (3)	0.1276 (3)	0.1321 (18)	
F10B	0.4066 (5)	0.9460 (4)	0.3969 (2)	0.144 (2)	
N7B	0.9133 (4)	0.7153 (3)	0.2348 (2)	0.0769 (13)	
H7BA	0.838777	0.692854	0.218678	0.092*	
H7BB	0.974374	0.708395	0.212741	0.092*	
N2A	0.9846 (6)	0.3040 (3)	0.5581 (2)	0.0906 (17)	
F9B	0.1368 (5)	0.8129 (3)	0.1428 (3)	0.151 (2)	
F10A	0.7786 (5)	0.2008 (4)	0.0839 (2)	0.152 (2)	
C6B	0.5592 (5)	0.6417 (3)	0.3082 (2)	0.0596 (12)	
C34B	1.0286 (5)	0.8401 (3)	0.3845 (3)	0.0686 (14)	
C10B	0.3079 (5)	0.4895 (3)	0.0592 (2)	0.0613 (12)	
N7A	0.5950 (4)	0.2383 (3)	0.2659 (3)	0.0834 (15)	
H7AA	0.669694	0.264523	0.275283	0.100*	
H7AB	0.538487	0.252597	0.289657	0.100*	
C24A	1.2785 (5)	0.3483 (3)	0.1986 (2)	0.0595 (12)	
C11A	1.0059 (5)	0.2480 (3)	0.4404 (2)	0.0574 (11)	
C20A	0.8694 (5)	0.3900 (3)	0.3675 (2)	0.0577 (11)	
C13B	0.6425 (5)	0.5702 (3)	0.1216 (2)	0.0598 (12)	
C11B	0.4216 (5)	0.5467 (3)	0.0907 (2)	0.0563 (11)	
C12B	0.5383 (5)	0.5250 (3)	0.0858 (2)	0.0611 (12)	
H12B	0.543429	0.477597	0.056555	0.073*	
C18A	1.0926 (5)	0.4187 (3)	0.3974 (2)	0.0582 (12)	
F1	0.5897 (9)	0.9650 (12)	0.4501 (4)	0.124 (5)	0.63 (3)
C35B	0.9339 (5)	0.7607 (3)	0.2937 (3)	0.0634 (13)	
C29B	0.9021 (5)	0.8249 (3)	0.3898 (3)	0.0689 (14)	
C14A	1.3417 (6)	0.2313 (4)	0.4111 (3)	0.0802 (17)	
C12A	1.1357 (5)	0.2532 (3)	0.4467 (2)	0.0634 (13)	
H12A	1.175822	0.271697	0.485641	0.076*	
F2AB	0.9328 (13)	−0.0599 (9)	0.0847 (7)	0.110 (6)	0.372 (17)
C25A	1.1581 (5)	0.3072 (3)	0.2020 (2)	0.0601 (12)	
C4B	0.3387 (4)	0.6124 (3)	0.2710 (2)	0.0557 (11)	
C10A	0.9385 (5)	0.2638 (3)	0.4945 (2)	0.0671 (13)	
C17A	1.2042 (5)	0.4766 (3)	0.4271 (2)	0.0616 (12)	
C18B	0.5010 (5)	0.7575 (3)	0.0781 (2)	0.0624 (12)	
N3A	1.2129 (6)	0.5575 (3)	0.4620 (3)	0.0966 (19)	
F12A	0.7437 (6)	0.2984 (4)	0.1512 (3)	0.168 (2)	
C3B	0.2158 (4)	0.5671 (3)	0.2678 (2)	0.0561 (11)	
C29A	0.5861 (5)	0.0807 (3)	0.1290 (3)	0.0676 (13)	
C5B	0.4388 (5)	0.6021 (3)	0.3008 (3)	0.0687 (14)	
H5B	0.422939	0.565993	0.316697	0.082*	
C34A	0.4632 (5)	0.0690 (3)	0.1429 (3)	0.0699 (14)	
C14B	0.7551 (6)	0.5349 (4)	0.1165 (3)	0.0818 (17)	
F1A	1.0823 (7)	0.0484 (4)	0.0981 (3)	0.188 (3)	
C25B	0.7455 (4)	0.8849 (3)	0.2883 (2)	0.0571 (11)	
C30B	0.8531 (6)	0.8547 (4)	0.4442 (3)	0.0820 (17)	
H30B	0.768261	0.844308	0.447195	0.098*	
C27A	0.9338 (5)	0.2678 (4)	0.1655 (3)	0.0734 (15)	
C19A	0.9731 (5)	0.4373 (3)	0.4035 (2)	0.0647 (13)	
H19A	0.965641	0.483856	0.433331	0.078*	
C26A	1.0534 (5)	0.3025 (4)	0.1643 (3)	0.0758 (16)	
H26A	1.067087	0.324692	0.136837	0.091*	
C20B	0.2997 (5)	0.7495 (3)	0.1199 (2)	0.0605 (12)	
C27B	0.5619 (5)	0.8853 (3)	0.3436 (3)	0.0686 (14)	
C26B	0.6804 (5)	0.9158 (3)	0.3394 (3)	0.0698 (14)	
H26B	0.720916	0.959462	0.372071	0.084*	
C33B	1.1119 (6)	0.8869 (4)	0.4340 (3)	0.0860 (18)	
H33B	1.196556	0.897808	0.430574	0.103*	
N1B	0.1805 (5)	0.5142 (3)	0.2923 (3)	0.0854 (15)	
C19B	0.3691 (5)	0.7469 (3)	0.0731 (3)	0.0688 (14)	
H19B	0.328507	0.737893	0.036018	0.083*	
C4A	0.7704 (4)	0.0652 (3)	0.2351 (2)	0.0574 (11)	
C3A	0.6486 (4)	0.0286 (3)	0.2447 (2)	0.0582 (12)	
N4A	1.3123 (6)	0.3900 (4)	0.1632 (3)	0.0997 (18)	
C7B	0.6554 (6)	0.6232 (4)	0.3431 (3)	0.0810 (18)	
C36A	0.3429 (5)	0.1421 (4)	0.2289 (3)	0.0885 (19)	
H36D	0.276676	0.139214	0.201342	0.133*	
H36E	0.359294	0.190963	0.262455	0.133*	
H36F	0.319454	0.103580	0.243693	0.133*	
C35A	0.5673 (5)	0.1770 (3)	0.2156 (3)	0.0666 (14)	
C21A	0.7519 (6)	0.4216 (4)	0.3717 (3)	0.0838 (18)	
C36B	1.1643 (5)	0.8002 (4)	0.2974 (3)	0.091 (2)	
H36A	1.206373	0.851605	0.307969	0.136*	
H36B	1.149268	0.773932	0.253944	0.136*	
H36C	1.214937	0.775642	0.312545	0.136*	
C6A	0.9483 (5)	0.0536 (3)	0.1751 (3)	0.0687 (14)	
C24B	0.8686 (5)	0.9275 (3)	0.2876 (3)	0.0621 (12)	
C5A	0.8308 (5)	0.0250 (3)	0.1843 (2)	0.0659 (14)	
H5A	0.789275	−0.022338	0.156203	0.079*	
C17B	0.5675 (6)	0.7679 (3)	0.0296 (3)	0.0724 (15)	
C22B	1.0668 (6)	0.9590 (5)	0.2541 (4)	0.103 (2)	
H22B	1.137304	0.957774	0.233238	0.124*	
C23B	1.0572 (7)	1.0185 (5)	0.3057 (4)	0.109 (3)	
H23B	1.120715	1.061329	0.321904	0.131*	
C7A	0.9960 (6)	0.0092 (5)	0.1129 (4)	0.093 (2)	
C21B	0.1628 (6)	0.7478 (4)	0.1095 (3)	0.0826 (18)	
C1B	−0.0073 (6)	0.5116 (4)	0.2455 (3)	0.090 (2)	
H1B	−0.091955	0.497384	0.233438	0.108*	
F12B	0.4659 (8)	0.8759 (4)	0.4272 (3)	0.183 (3)	
C31B	0.9372 (8)	0.9018 (4)	0.4950 (3)	0.098 (2)	
H31B	0.907970	0.923042	0.532864	0.117*	
C15A	1.4191 (7)	0.5431 (4)	0.4589 (4)	0.098 (2)	
H15A	1.504818	0.557759	0.467270	0.118*	
C22A	1.5008 (6)	0.4025 (4)	0.2139 (3)	0.093 (2)	
H22A	1.585440	0.419019	0.225868	0.112*	
C16A	1.3468 (7)	0.5895 (4)	0.4772 (4)	0.099 (2)	
H16A	1.377127	0.641109	0.499196	0.118*	
F3A	1.0667 (7)	−0.0332 (5)	0.1208 (3)	0.187 (3)	
C33A	0.3795 (6)	0.0031 (4)	0.1087 (3)	0.0894 (19)	
H33A	0.297934	−0.005385	0.117875	0.107*	
C28B	0.5080 (7)	0.9233 (5)	0.4040 (4)	0.108 (3)	
C2B	0.0502 (6)	0.4834 (4)	0.2772 (3)	0.0876 (19)	
H2B	0.008803	0.446255	0.288707	0.105*	
C30A	0.6271 (6)	0.0289 (5)	0.0796 (3)	0.090 (2)	
H30A	0.707705	0.037819	0.069289	0.109*	
C9A	0.8822 (8)	0.3062 (5)	0.5927 (4)	0.103 (2)	
H9A	0.891527	0.330723	0.634949	0.124*	
C2A	0.4601 (6)	−0.0515 (4)	0.2364 (4)	0.089 (2)	
H2A	0.394852	−0.093447	0.221192	0.107*	
C8A	0.7760 (8)	0.2726 (5)	0.5631 (4)	0.105 (2)	
H8A	0.702999	0.268986	0.581573	0.126*	
C9B	0.1616 (8)	0.3787 (5)	0.0056 (4)	0.112 (3)	
H9B	0.124564	0.328493	−0.018356	0.134*	
C32B	1.0626 (8)	0.9171 (4)	0.4895 (4)	0.102 (2)	
H32B	1.116098	0.948625	0.524102	0.122*	
C28A	0.8333 (8)	0.2683 (6)	0.1204 (5)	0.113 (3)	
C8B	0.0918 (8)	0.4290 (5)	0.0290 (4)	0.111 (3)	
H8B	0.005704	0.416605	0.022696	0.133*	
C16B	0.6234 (10)	0.7777 (5)	−0.0603 (4)	0.122 (3)	
H16B	0.619349	0.776135	−0.099253	0.146*	
C1A	0.4548 (6)	0.0097 (4)	0.2884 (4)	0.090 (2)	
H1A	0.386990	0.013232	0.311242	0.108*	
C31A	0.5419 (8)	−0.0377 (5)	0.0454 (3)	0.106 (2)	
H31A	0.566820	−0.074653	0.011940	0.128*	
C23A	1.4433 (7)	0.4179 (5)	0.1731 (4)	0.101 (2)	
H23A	1.485172	0.444757	0.152665	0.122*	
C32A	0.4230 (8)	−0.0503 (5)	0.0596 (4)	0.108 (2)	
H32A	0.369305	−0.095791	0.035894	0.130*	
C15B	0.7321 (10)	0.8030 (6)	−0.0242 (4)	0.125 (3)	
H15B	0.807272	0.818666	−0.036936	0.150*	
F2AA	0.9169 (7)	−0.0177 (6)	0.0666 (3)	0.110 (4)	0.628 (17)
F2	0.5666 (17)	0.9970 (9)	0.4361 (8)	0.095 (6)	0.37 (3)
F8BA	0.1224 (13)	0.7464 (7)	0.0584 (6)	0.093 (3)	0.5
F3	0.8822 (13)	0.2848 (8)	0.0749 (5)	0.122 (4)	0.5
F4	0.8672 (11)	0.3240 (7)	0.1013 (6)	0.106 (3)	0.5

### 2-Amino-1-methylbenzimidazolium tetrakis[4,4,4-trifluoro-1-(1,3-thiazol-2-yl)butane-1,3-dionato-κ2O,O']cerium(III) Atomic displacement parameters (Å^2^)

**Table:**

	*U*^11^	*U*^22^	*U*^33^	*U*^12^	*U*^13^	*U*^23^
Ce1A	0.03851 (14)	0.05367 (15)	0.05173 (16)	0.00509 (10)	−0.00116 (10)	0.02051 (12)
Ce1B	0.04195 (15)	0.05040 (15)	0.06459 (18)	0.00317 (10)	−0.00931 (11)	0.02377 (13)
S1B	0.0556 (8)	0.1049 (12)	0.1013 (12)	0.0038 (8)	−0.0095 (8)	0.0655 (10)
S4A	0.0593 (8)	0.1244 (14)	0.1071 (13)	0.0003 (9)	−0.0040 (8)	0.0754 (12)
S3A	0.0656 (9)	0.0878 (11)	0.1174 (15)	0.0014 (8)	−0.0150 (9)	0.0319 (10)
S1A	0.0675 (9)	0.0784 (10)	0.1134 (14)	0.0076 (8)	0.0178 (9)	0.0337 (10)
S2A	0.0808 (12)	0.1260 (16)	0.1118 (15)	0.0325 (11)	0.0357 (11)	0.0548 (13)
S2B	0.0817 (12)	0.1186 (16)	0.1196 (16)	0.0036 (11)	−0.0058 (11)	0.0139 (13)
S3B	0.0982 (14)	0.1175 (15)	0.1189 (16)	0.0210 (12)	0.0284 (12)	0.0394 (13)
N4B	0.0230 (13)	0.0241 (12)	0.0410 (16)	−0.0126 (10)	−0.0083 (11)	0.0038 (11)
S4B	0.1085 (17)	0.135 (2)	0.206 (3)	0.0196 (15)	0.0231 (18)	0.080 (2)
O4A	0.0474 (17)	0.0620 (19)	0.0558 (19)	0.0146 (14)	0.0017 (14)	0.0205 (16)
O2B	0.0447 (17)	0.075 (2)	0.077 (2)	0.0075 (16)	−0.0080 (16)	0.040 (2)
O6A	0.0524 (19)	0.060 (2)	0.069 (2)	0.0102 (15)	−0.0088 (16)	0.0186 (17)
O4B	0.0536 (19)	0.062 (2)	0.072 (2)	0.0092 (16)	−0.0092 (17)	0.0207 (18)
O7B	0.0455 (17)	0.0557 (19)	0.069 (2)	0.0000 (14)	−0.0069 (15)	0.0182 (16)
O3A	0.0470 (18)	0.087 (2)	0.057 (2)	0.0092 (17)	0.0054 (15)	0.0286 (18)
O6B	0.0511 (18)	0.065 (2)	0.060 (2)	0.0142 (15)	−0.0032 (15)	0.0243 (16)
O2A	0.0466 (17)	0.0523 (18)	0.065 (2)	0.0038 (14)	0.0007 (15)	0.0133 (15)
O8B	0.0506 (19)	0.070 (2)	0.069 (2)	0.0020 (16)	−0.0003 (17)	0.0208 (18)
O1A	0.0474 (19)	0.074 (2)	0.065 (2)	0.0096 (17)	0.0021 (16)	0.0090 (18)
O7A	0.051 (2)	0.102 (3)	0.083 (3)	0.0041 (19)	−0.0001 (18)	0.061 (2)
O8A	0.0452 (18)	0.095 (3)	0.065 (2)	0.0104 (17)	−0.0010 (16)	0.041 (2)
O5B	0.0531 (19)	0.076 (2)	0.066 (2)	0.0065 (17)	−0.0042 (17)	0.0290 (19)
F6A	0.057 (2)	0.168 (4)	0.125 (3)	0.003 (2)	0.010 (2)	0.059 (3)
N2B	0.064 (3)	0.056 (2)	0.054 (2)	−0.0248 (19)	−0.0245 (19)	0.0182 (19)
O1B	0.051 (2)	0.082 (3)	0.105 (3)	0.0006 (17)	−0.0116 (19)	0.060 (2)
N6B	0.049 (2)	0.073 (3)	0.079 (3)	0.010 (2)	−0.006 (2)	0.031 (2)
F5A	0.072 (2)	0.200 (5)	0.087 (3)	0.033 (3)	−0.022 (2)	0.029 (3)
F1B	0.079 (2)	0.157 (4)	0.146 (4)	0.064 (3)	0.017 (2)	0.082 (3)
F8A	0.096 (3)	0.090 (3)	0.117 (3)	0.044 (2)	−0.011 (2)	0.003 (2)
F4B	0.063 (2)	0.144 (4)	0.138 (4)	0.023 (2)	0.014 (2)	0.020 (3)
O3B	0.059 (2)	0.052 (2)	0.097 (3)	0.0029 (16)	−0.022 (2)	0.0107 (19)
F3B	0.105 (3)	0.146 (4)	0.090 (3)	0.018 (3)	−0.033 (2)	0.051 (3)
O5A	0.053 (2)	0.053 (2)	0.100 (3)	0.0057 (16)	−0.0125 (19)	0.017 (2)
F7A	0.137 (4)	0.145 (4)	0.112 (3)	0.077 (3)	−0.026 (3)	0.040 (3)
F5B	0.090 (3)	0.119 (3)	0.122 (3)	0.048 (2)	−0.001 (2)	−0.004 (3)
F9A	0.067 (2)	0.121 (3)	0.133 (4)	0.025 (2)	0.014 (2)	0.012 (3)
N5B	0.043 (2)	0.080 (3)	0.083 (3)	0.008 (2)	−0.010 (2)	0.040 (3)
F6B	0.141 (4)	0.176 (4)	0.131 (4)	0.087 (3)	0.001 (3)	0.076 (3)
F2B	0.094 (3)	0.150 (4)	0.185 (4)	0.022 (3)	−0.016 (3)	0.125 (4)
F4A	0.097 (3)	0.126 (4)	0.163 (4)	0.061 (3)	−0.007 (3)	0.041 (3)
N6A	0.044 (2)	0.080 (3)	0.088 (3)	0.014 (2)	0.005 (2)	0.031 (3)
N5A	0.035 (2)	0.095 (3)	0.079 (3)	0.008 (2)	−0.004 (2)	0.041 (3)
C13A	0.047 (2)	0.050 (2)	0.056 (3)	0.0083 (19)	−0.006 (2)	0.017 (2)
F8BB	0.071 (5)	0.125 (8)	0.092 (6)	0.011 (6)	−0.022 (4)	0.027 (6)
N3B	0.094 (4)	0.098 (4)	0.043 (2)	0.037 (3)	0.019 (2)	0.023 (2)
N1A	0.043 (2)	0.073 (3)	0.099 (3)	0.0068 (19)	−0.007 (2)	0.045 (3)
F7B	0.065 (2)	0.181 (5)	0.148 (4)	−0.005 (3)	0.002 (3)	0.079 (4)
F10B	0.111 (4)	0.168 (5)	0.126 (4)	0.050 (3)	0.043 (3)	0.023 (3)
N7B	0.049 (2)	0.083 (3)	0.087 (3)	0.007 (2)	−0.010 (2)	0.026 (3)
N2A	0.117 (4)	0.114 (4)	0.060 (3)	0.066 (4)	0.023 (3)	0.036 (3)
F9B	0.098 (3)	0.152 (4)	0.179 (5)	0.074 (3)	−0.008 (3)	0.022 (4)
F10A	0.125 (4)	0.196 (5)	0.113 (4)	−0.021 (4)	−0.056 (3)	0.068 (4)
C6B	0.048 (3)	0.067 (3)	0.068 (3)	0.018 (2)	−0.002 (2)	0.030 (3)
C34B	0.055 (3)	0.064 (3)	0.085 (4)	0.010 (2)	−0.006 (3)	0.030 (3)
C10B	0.059 (3)	0.060 (3)	0.058 (3)	0.003 (2)	−0.004 (2)	0.021 (2)
N7A	0.052 (3)	0.078 (3)	0.100 (4)	0.011 (2)	−0.008 (3)	0.019 (3)
C24A	0.058 (3)	0.062 (3)	0.061 (3)	0.006 (2)	0.005 (2)	0.030 (2)
C11A	0.060 (3)	0.061 (3)	0.051 (3)	0.011 (2)	0.005 (2)	0.024 (2)
C20A	0.057 (3)	0.061 (3)	0.058 (3)	0.015 (2)	0.000 (2)	0.026 (2)
C13B	0.058 (3)	0.069 (3)	0.057 (3)	0.011 (2)	0.004 (2)	0.030 (2)
C11B	0.061 (3)	0.053 (3)	0.054 (3)	0.003 (2)	−0.005 (2)	0.025 (2)
C12B	0.055 (3)	0.062 (3)	0.062 (3)	0.006 (2)	0.002 (2)	0.023 (2)
C18A	0.059 (3)	0.055 (3)	0.060 (3)	0.003 (2)	−0.007 (2)	0.027 (2)
F1	0.098 (5)	0.154 (9)	0.077 (5)	0.011 (5)	−0.004 (4)	0.010 (5)
C35B	0.044 (3)	0.065 (3)	0.079 (4)	0.009 (2)	−0.010 (2)	0.029 (3)
C29B	0.058 (3)	0.070 (3)	0.081 (4)	0.013 (3)	−0.006 (3)	0.034 (3)
C14A	0.057 (3)	0.096 (5)	0.071 (4)	0.016 (3)	−0.003 (3)	0.018 (3)
C12A	0.056 (3)	0.073 (3)	0.056 (3)	0.010 (2)	−0.005 (2)	0.023 (2)
F2AB	0.100 (6)	0.096 (7)	0.101 (6)	0.013 (4)	0.022 (4)	0.011 (4)
C25A	0.060 (3)	0.065 (3)	0.057 (3)	0.012 (2)	0.006 (2)	0.027 (2)
C4B	0.050 (3)	0.060 (3)	0.061 (3)	0.013 (2)	0.000 (2)	0.029 (2)
C10A	0.074 (3)	0.076 (3)	0.058 (3)	0.030 (3)	0.017 (3)	0.028 (3)
C17A	0.064 (3)	0.053 (3)	0.063 (3)	0.000 (2)	−0.012 (2)	0.025 (2)
C18B	0.062 (3)	0.053 (3)	0.063 (3)	0.011 (2)	0.001 (2)	0.016 (2)
N3A	0.103 (4)	0.068 (3)	0.097 (4)	−0.024 (3)	−0.033 (3)	0.031 (3)
F12A	0.118 (4)	0.228 (6)	0.195 (5)	0.067 (4)	−0.016 (4)	0.112 (5)
C3B	0.053 (3)	0.064 (3)	0.058 (3)	0.016 (2)	0.005 (2)	0.030 (2)
C29A	0.054 (3)	0.086 (4)	0.068 (3)	0.019 (3)	−0.004 (2)	0.035 (3)
C5B	0.057 (3)	0.077 (3)	0.085 (4)	0.013 (3)	0.000 (3)	0.047 (3)
C34A	0.051 (3)	0.080 (4)	0.078 (4)	0.015 (3)	−0.009 (3)	0.032 (3)
C14B	0.065 (4)	0.097 (5)	0.080 (4)	0.028 (3)	0.003 (3)	0.027 (4)
F1A	0.201 (6)	0.191 (6)	0.125 (4)	0.016 (5)	0.084 (4)	0.027 (4)
C25B	0.047 (2)	0.057 (3)	0.066 (3)	0.004 (2)	−0.009 (2)	0.028 (2)
C30B	0.077 (4)	0.082 (4)	0.093 (5)	0.026 (3)	0.009 (3)	0.037 (4)
C27A	0.062 (3)	0.097 (4)	0.067 (3)	0.008 (3)	−0.006 (3)	0.045 (3)
C19A	0.063 (3)	0.059 (3)	0.064 (3)	0.011 (2)	−0.004 (2)	0.018 (2)
C26A	0.060 (3)	0.098 (4)	0.082 (4)	−0.001 (3)	−0.007 (3)	0.058 (4)
C20B	0.050 (3)	0.057 (3)	0.068 (3)	0.011 (2)	−0.008 (2)	0.019 (2)
C27B	0.060 (3)	0.072 (3)	0.063 (3)	0.006 (3)	0.000 (2)	0.020 (3)
C26B	0.052 (3)	0.068 (3)	0.068 (3)	−0.001 (2)	−0.007 (2)	0.014 (3)
C33B	0.063 (4)	0.083 (4)	0.099 (5)	0.003 (3)	−0.014 (3)	0.029 (4)
N1B	0.068 (3)	0.097 (4)	0.099 (4)	0.010 (3)	0.012 (3)	0.052 (3)
C19B	0.054 (3)	0.087 (4)	0.060 (3)	0.014 (3)	−0.009 (2)	0.025 (3)
C4A	0.047 (2)	0.055 (3)	0.063 (3)	0.008 (2)	−0.008 (2)	0.019 (2)
C3A	0.047 (2)	0.051 (2)	0.073 (3)	0.005 (2)	−0.006 (2)	0.024 (2)
N4A	0.084 (4)	0.104 (4)	0.120 (5)	−0.004 (3)	0.006 (3)	0.066 (4)
C7B	0.062 (3)	0.098 (5)	0.108 (5)	0.020 (3)	−0.003 (3)	0.067 (4)
C36A	0.050 (3)	0.095 (5)	0.116 (5)	0.019 (3)	0.019 (3)	0.038 (4)
C35A	0.040 (2)	0.073 (3)	0.083 (4)	0.012 (2)	−0.006 (2)	0.029 (3)
C21A	0.073 (4)	0.082 (4)	0.082 (4)	0.029 (3)	−0.008 (3)	0.014 (3)
C36B	0.044 (3)	0.105 (5)	0.106 (5)	0.004 (3)	−0.005 (3)	0.032 (4)
C6A	0.052 (3)	0.076 (3)	0.062 (3)	0.020 (3)	−0.005 (2)	0.009 (3)
C24B	0.045 (3)	0.060 (3)	0.078 (3)	0.005 (2)	−0.006 (2)	0.028 (3)
C5A	0.047 (3)	0.061 (3)	0.068 (3)	0.009 (2)	−0.006 (2)	0.006 (2)
C17B	0.073 (4)	0.073 (3)	0.063 (3)	0.022 (3)	0.012 (3)	0.017 (3)
C22B	0.056 (4)	0.101 (5)	0.160 (8)	0.007 (4)	0.014 (4)	0.067 (6)
C23B	0.087 (5)	0.082 (5)	0.140 (7)	−0.023 (4)	−0.035 (5)	0.047 (5)
C7A	0.064 (3)	0.096 (4)	0.094 (4)	0.020 (3)	0.009 (3)	0.011 (3)
C21B	0.056 (3)	0.106 (5)	0.079 (4)	0.016 (3)	−0.005 (3)	0.031 (4)
C1B	0.055 (3)	0.124 (6)	0.098 (5)	−0.003 (3)	−0.001 (3)	0.064 (4)
F12B	0.229 (6)	0.201 (6)	0.124 (4)	0.031 (5)	0.076 (4)	0.077 (4)
C31B	0.104 (6)	0.098 (5)	0.081 (5)	0.027 (4)	−0.005 (4)	0.024 (4)
C15A	0.081 (5)	0.093 (5)	0.114 (6)	−0.019 (4)	−0.027 (4)	0.051 (4)
C22A	0.063 (4)	0.106 (5)	0.111 (5)	−0.008 (3)	0.003 (4)	0.056 (4)
C16A	0.089 (5)	0.061 (4)	0.129 (6)	−0.010 (3)	−0.035 (4)	0.035 (4)
F3A	0.184 (4)	0.191 (4)	0.185 (4)	0.091 (4)	0.058 (4)	0.055 (3)
C33A	0.069 (4)	0.090 (5)	0.101 (5)	0.012 (3)	−0.014 (3)	0.033 (4)
C28B	0.074 (5)	0.121 (6)	0.083 (5)	−0.006 (4)	0.013 (4)	0.006 (4)
C2B	0.075 (4)	0.104 (5)	0.097 (5)	0.001 (4)	0.011 (4)	0.063 (4)
C30A	0.074 (4)	0.130 (6)	0.077 (4)	0.037 (4)	0.004 (3)	0.046 (4)
C9A	0.119 (4)	0.111 (4)	0.087 (4)	0.044 (3)	0.025 (3)	0.040 (3)
C2A	0.059 (3)	0.073 (4)	0.136 (6)	−0.008 (3)	−0.012 (4)	0.053 (4)
C8A	0.107 (4)	0.113 (4)	0.107 (4)	0.040 (3)	0.047 (3)	0.049 (3)
C9B	0.091 (5)	0.078 (5)	0.131 (7)	−0.016 (4)	−0.039 (5)	0.022 (5)
C32B	0.094 (5)	0.084 (5)	0.102 (6)	0.008 (4)	−0.021 (4)	0.019 (4)
C28A	0.077 (5)	0.166 (9)	0.124 (7)	0.012 (5)	−0.006 (5)	0.095 (7)
C8B	0.078 (5)	0.097 (5)	0.126 (7)	−0.012 (4)	−0.014 (4)	0.027 (5)
C16B	0.165 (9)	0.124 (7)	0.073 (5)	0.045 (7)	0.034 (6)	0.030 (5)
C1A	0.065 (4)	0.082 (4)	0.127 (6)	0.007 (3)	0.020 (4)	0.050 (4)
C31A	0.112 (6)	0.119 (6)	0.077 (5)	0.046 (5)	−0.011 (4)	0.021 (4)
C23A	0.082 (4)	0.120 (6)	0.119 (6)	−0.021 (4)	−0.003 (4)	0.085 (5)
C32A	0.104 (6)	0.095 (5)	0.101 (6)	0.013 (4)	−0.025 (5)	0.019 (4)
C15B	0.141 (8)	0.133 (7)	0.102 (6)	0.038 (6)	0.071 (6)	0.045 (6)
F2AA	0.091 (4)	0.140 (5)	0.073 (4)	0.032 (3)	0.003 (3)	0.014 (3)
F2	0.091 (7)	0.087 (7)	0.087 (7)	0.010 (4)	0.018 (4)	0.020 (4)
F8BA	0.073 (5)	0.130 (8)	0.089 (6)	0.032 (6)	−0.016 (4)	0.053 (6)
F3	0.124 (7)	0.156 (8)	0.101 (7)	0.005 (6)	−0.021 (5)	0.079 (6)
F4	0.099 (6)	0.124 (7)	0.119 (7)	0.017 (5)	−0.020 (5)	0.078 (6)

### 2-Amino-1-methylbenzimidazolium tetrakis[4,4,4-trifluoro-1-(1,3-thiazol-2-yl)butane-1,3-dionato-κ2O,O']cerium(III) Geometric parameters (Å, º)

**Table:**

Ce1A—O2A	2.405 (3)	C24A—C25A	1.442 (7)
Ce1A—O7A	2.436 (3)	C11A—C12A	1.418 (7)
Ce1A—O5A	2.443 (4)	C11A—C10A	1.461 (7)
Ce1A—O3A	2.443 (3)	C20A—C19A	1.364 (7)
Ce1A—O1A	2.457 (4)	C20A—C21A	1.526 (8)
Ce1A—O4A	2.467 (3)	C13B—C12B	1.356 (7)
Ce1A—O6A	2.516 (4)	C13B—C14B	1.516 (8)
Ce1A—O8A	2.580 (3)	C11B—C12B	1.421 (7)
Ce1B—O7B	2.434 (3)	C12B—H12B	0.9300
Ce1B—O3B	2.444 (4)	C18A—C19A	1.426 (7)
Ce1B—O6B	2.453 (3)	C18A—C17A	1.459 (7)
Ce1B—O1B	2.453 (4)	F1—C28B	1.301 (11)
Ce1B—O8B	2.460 (4)	C29B—C30B	1.369 (9)
Ce1B—O5B	2.476 (4)	C12A—H12A	0.9300
Ce1B—O4B	2.524 (4)	F2AB—C7A	1.302 (14)
Ce1B—O2B	2.543 (3)	F2AB—F3A	1.591 (17)
S1B—C1B	1.702 (6)	C25A—C26A	1.429 (7)
S1B—C3B	1.716 (5)	C4B—C5B	1.401 (7)
S4A—C22A	1.694 (6)	C4B—C3B	1.462 (7)
S4A—C24A	1.705 (5)	C17A—N3A	1.459 (7)
S3A—C17A	1.696 (6)	C18B—C19B	1.427 (7)
S3A—C15A	1.715 (7)	C18B—C17B	1.453 (8)
S1A—C1A	1.663 (6)	N3A—C16A	1.471 (9)
S1A—C3A	1.691 (6)	F12A—C28A	1.328 (11)
S2A—C10A	1.705 (6)	C3B—N1B	1.395 (7)
S2A—C8A	1.735 (8)	C29A—C30A	1.370 (9)
S2B—C8B	1.661 (8)	C29A—C34A	1.399 (7)
S2B—C10B	1.689 (6)	C5B—H5B	0.9300
S3B—C15B	1.608 (9)	C34A—C33A	1.377 (8)
S3B—C17B	1.673 (6)	F1A—C7A	1.260 (9)
N4B—C23B	1.567 (9)	C25B—C26B	1.410 (8)
N4B—C24B	1.641 (6)	C25B—C24B	1.462 (7)
S4B—C22B	1.593 (7)	C30B—C31B	1.400 (9)
S4B—C24B	1.622 (6)	C30B—H30B	0.9300
O4A—C13A	1.248 (6)	C27A—C26A	1.375 (8)
O2B—C6B	1.258 (6)	C27A—C28A	1.548 (9)
O6A—C20A	1.270 (6)	C19A—H19A	0.9300
O4B—C13B	1.269 (6)	C26A—H26A	0.9300
O7B—C25B	1.260 (6)	C20B—C19B	1.362 (8)
O3A—C11A	1.260 (6)	C20B—C21B	1.519 (8)
O6B—C20B	1.272 (6)	C27B—C26B	1.356 (7)
O2A—C4A	1.252 (6)	C27B—C28B	1.532 (9)
O8B—C27B	1.263 (6)	C26B—H26B	0.9300
O1A—C6A	1.260 (6)	C33B—C32B	1.394 (10)
O7A—C25A	1.248 (6)	C33B—H33B	0.9300
O8A—C27A	1.253 (6)	N1B—C2B	1.432 (8)
O5B—C18B	1.256 (6)	C19B—H19B	0.9300
F6A—C14A	1.316 (8)	C4A—C5A	1.418 (7)
N2B—C9B	1.544 (9)	C4A—C3A	1.469 (7)
N2B—C10B	1.596 (7)	N4A—C23A	1.427 (9)
O1B—C4B	1.259 (6)	C36A—H36D	0.9600
N6B—C35B	1.350 (6)	C36A—H36E	0.9600
N6B—C34B	1.381 (7)	C36A—H36F	0.9600
N6B—C36B	1.469 (7)	C36B—H36A	0.9600
F5A—C14A	1.324 (7)	C36B—H36B	0.9600
F1B—C7B	1.314 (8)	C36B—H36C	0.9600
F8A—C21A	1.337 (7)	C6A—C5A	1.371 (7)
F4B—C14B	1.322 (8)	C6A—C7A	1.560 (9)
O3B—C11B	1.233 (6)	C5A—H5A	0.9300
F3B—C7B	1.351 (8)	C22B—C23B	1.346 (11)
O5A—C18A	1.242 (6)	C22B—H22B	0.9300
F7A—C21A	1.325 (8)	C23B—H23B	0.9300
F5B—C14B	1.328 (8)	C7A—F2AA	1.272 (10)
F9A—C21A	1.324 (8)	C7A—F3A	1.308 (10)
N5B—C35B	1.320 (7)	C21B—F8BA	1.296 (14)
N5B—C29B	1.395 (7)	C1B—C2B	1.327 (9)
N5B—H5BA	0.8600	C1B—H1B	0.9300
F6B—C14B	1.310 (8)	F12B—C28B	1.293 (11)
F2B—C7B	1.325 (7)	C31B—C32B	1.379 (10)
F4A—C14A	1.310 (8)	C31B—H31B	0.9300
N6A—C35A	1.346 (7)	C15A—C16A	1.274 (11)
N6A—C34A	1.400 (8)	C15A—H15A	0.9300
N6A—C36A	1.466 (7)	C22A—C23A	1.327 (10)
N5A—C35A	1.336 (7)	C22A—H22A	0.9300
N5A—C29A	1.388 (7)	C16A—H16A	0.9300
N5A—H5AA	0.8600	C33A—C32A	1.392 (11)
C13A—C12A	1.372 (7)	C33A—H33A	0.9300
C13A—C14A	1.542 (7)	C28B—F2	1.366 (15)
F8BB—C21B	1.340 (15)	C2B—H2B	0.9300
N3B—C16B	1.484 (10)	C30A—C31A	1.393 (11)
N3B—C17B	1.542 (7)	C30A—H30A	0.9300
N1A—C2A	1.463 (8)	C9A—C8A	1.278 (11)
N1A—C3A	1.503 (6)	C9A—H9A	0.9300
F7B—C21B	1.328 (8)	C2A—C1A	1.358 (10)
F10B—C28B	1.313 (10)	C2A—H2A	0.9300
N7B—C35B	1.330 (7)	C8A—H8A	0.9300
N7B—H7BA	0.8600	C9B—C8B	1.320 (11)
N7B—H7BB	0.8600	C9B—H9B	0.9300
N2A—C9A	1.410 (9)	C32B—H32B	0.9300
N2A—C10A	1.447 (8)	C28A—F4	1.351 (14)
F9B—C21B	1.300 (8)	C28A—F3	1.359 (15)
F10A—C28A	1.288 (11)	C8B—H8B	0.9300
C6B—C5B	1.377 (7)	C16B—C15B	1.358 (13)
C6B—C7B	1.529 (7)	C16B—H16B	0.9300
C34B—C33B	1.376 (8)	C1A—H1A	0.9300
C34B—C29B	1.390 (8)	C31A—C32A	1.362 (11)
C10B—C11B	1.477 (7)	C31A—H31A	0.9300
N7A—C35A	1.305 (7)	C23A—H23A	0.9300
N7A—H7AA	0.8600	C32A—H32A	0.9300
N7A—H7AB	0.8600	C15B—H15B	0.9300
C24A—N4A	1.419 (7)		
			
Cg2···Cg10	3.668 (4)	Cg9···Cg12	3.999 (4)
Cg3···Cg13^i^	3.772 (5)	Cg10···Cg2	3.668 (4)
			
O2A—Ce1A—O7A	139.60 (13)	F6B—C14B—C13B	110.0 (5)
O2A—Ce1A—O5A	145.78 (13)	F4B—C14B—C13B	112.9 (6)
O7A—Ce1A—O5A	72.68 (15)	F5B—C14B—C13B	115.0 (5)
O2A—Ce1A—O3A	72.92 (12)	O7B—C25B—C26B	123.7 (5)
O7A—Ce1A—O3A	146.30 (12)	O7B—C25B—C24B	118.6 (5)
O5A—Ce1A—O3A	78.70 (14)	C26B—C25B—C24B	117.7 (5)
O1A—Ce1A—O2A	70.26 (11)	C29B—C30B—C31B	116.7 (6)
O7A—Ce1A—O1A	77.21 (14)	C29B—C30B—H30B	121.7
O5A—Ce1A—O1A	142.46 (12)	C31B—C30B—H30B	121.7
O3A—Ce1A—O1A	119.21 (14)	O8A—C27A—C26A	128.9 (5)
O2A—Ce1A—O4A	112.75 (12)	O8A—C27A—C28A	114.2 (5)
O7A—Ce1A—O4A	85.24 (12)	C26A—C27A—C28A	116.9 (5)
O5A—Ce1A—O4A	73.31 (12)	C20A—C19A—C18A	122.9 (5)
O3A—Ce1A—O4A	69.57 (11)	C20A—C19A—H19A	118.5
O1A—Ce1A—O4A	82.43 (12)	C18A—C19A—H19A	118.5
O2A—Ce1A—O6A	86.93 (11)	C27A—C26A—C25A	124.9 (5)
O7A—Ce1A—O6A	106.36 (13)	C27A—C26A—H26A	117.6
O5A—Ce1A—O6A	68.84 (12)	C25A—C26A—H26A	117.6
O3A—Ce1A—O6A	78.67 (12)	O6B—C20B—C19B	129.4 (5)
O1A—Ce1A—O6A	142.87 (12)	O6B—C20B—C21B	114.1 (5)
O4A—Ce1A—O6A	134.33 (11)	C19B—C20B—C21B	116.3 (5)
O2A—Ce1A—O8A	80.20 (12)	O8B—C27B—C26B	129.6 (5)
O7A—Ce1A—O8A	68.99 (11)	O8B—C27B—C28B	113.7 (5)
O5A—Ce1A—O8A	112.29 (13)	C26B—C27B—C28B	116.7 (5)
O3A—Ce1A—O8A	140.54 (11)	C27B—C26B—C25B	123.6 (5)
O1A—Ce1A—O8A	75.95 (13)	C27B—C26B—H26B	118.2
O4A—Ce1A—O8A	149.28 (11)	C25B—C26B—H26B	118.2
O6A—Ce1A—O8A	71.45 (12)	C34B—C33B—C32B	116.4 (6)
O7B—Ce1B—O3B	144.05 (14)	C34B—C33B—H33B	121.8
O7B—Ce1B—O6B	117.73 (12)	C32B—C33B—H33B	121.8
O3B—Ce1B—O6B	73.70 (12)	C3B—N1B—C2B	109.1 (5)
O7B—Ce1B—O1B	141.02 (13)	C20B—C19B—C18B	123.2 (5)
O3B—Ce1B—O1B	71.90 (15)	C20B—C19B—H19B	118.4
O6B—Ce1B—O1B	80.03 (12)	C18B—C19B—H19B	118.4
O7B—Ce1B—O8B	70.40 (12)	O2A—C4A—C5A	123.6 (5)
O3B—Ce1B—O8B	144.61 (13)	O2A—C4A—C3A	117.1 (5)
O6B—Ce1B—O8B	81.63 (12)	C5A—C4A—C3A	119.2 (4)
O1B—Ce1B—O8B	79.25 (13)	C4A—C3A—N1A	128.1 (5)
O7B—Ce1B—O5B	74.43 (12)	C4A—C3A—S1A	117.6 (4)
O3B—Ce1B—O5B	79.07 (14)	N1A—C3A—S1A	114.3 (4)
O5B—Ce1B—O6B	69.86 (12)	C24A—N4A—C23A	108.7 (5)
O1B—Ce1B—O5B	142.90 (12)	F1B—C7B—F2B	107.4 (6)
O8B—Ce1B—O5B	115.84 (13)	F1B—C7B—F3B	105.5 (5)
O7B—Ce1B—O4B	83.49 (11)	F2B—C7B—F3B	106.6 (6)
O3B—Ce1B—O4B	68.95 (12)	F1B—C7B—C6B	111.9 (5)
O6B—Ce1B—O4B	136.58 (11)	F2B—C7B—C6B	114.8 (5)
O1B—Ce1B—O4B	107.72 (13)	F3B—C7B—C6B	110.0 (5)
O8B—Ce1B—O4B	141.47 (12)	N6A—C36A—H36D	109.5
O5B—Ce1B—O4B	81.89 (13)	N6A—C36A—H36E	109.5
O7B—Ce1B—O2B	80.22 (12)	H36D—C36A—H36E	109.5
O3B—Ce1B—O2B	110.36 (13)	N6A—C36A—H36F	109.5
O6B—Ce1B—O2B	145.01 (12)	H36D—C36A—H36F	109.5
O1B—Ce1B—O2B	69.23 (11)	H36E—C36A—H36F	109.5
O8B—Ce1B—O2B	76.70 (13)	N7A—C35A—N5A	125.8 (5)
O5B—Ce1B—O2B	144.79 (12)	N7A—C35A—N6A	125.2 (5)
O4B—Ce1B—O2B	71.19 (12)	N5A—C35A—N6A	109.0 (5)
C1B—S1B—C3B	91.8 (3)	F9A—C21A—F7A	104.9 (6)
C22A—S4A—C24A	91.9 (3)	F9A—C21A—F8A	106.2 (6)
C17A—S3A—C15A	90.9 (4)	F7A—C21A—F8A	107.8 (6)
C1A—S1A—C3A	92.7 (3)	F9A—C21A—C20A	112.4 (6)
C10A—S2A—C8A	90.2 (4)	F7A—C21A—C20A	109.8 (6)
C8B—S2B—C10B	92.2 (4)	F8A—C21A—C20A	115.1 (5)
C15B—S3B—C17B	94.1 (5)	N6B—C36B—H36A	109.5
C23B—N4B—C24B	96.1 (4)	N6B—C36B—H36B	109.5
C22B—S4B—C24B	96.5 (4)	H36A—C36B—H36B	109.5
C13A—O4A—Ce1A	129.0 (3)	N6B—C36B—H36C	109.5
C6B—O2B—Ce1B	130.5 (3)	H36A—C36B—H36C	109.5
C20A—O6A—Ce1A	132.9 (3)	H36B—C36B—H36C	109.5
C13B—O4B—Ce1B	132.3 (3)	O1A—C6A—C5A	129.8 (5)
C25B—O7B—Ce1B	138.3 (3)	O1A—C6A—C7A	113.3 (5)
C11A—O3A—Ce1A	133.6 (3)	C5A—C6A—C7A	116.8 (5)
C20B—O6B—Ce1B	129.4 (3)	C25B—C24B—S4B	118.7 (4)
C4A—O2A—Ce1A	139.7 (3)	C25B—C24B—N4B	126.6 (4)
C27B—O8B—Ce1B	132.9 (4)	S4B—C24B—N4B	114.7 (3)
C6A—O1A—Ce1A	132.0 (3)	C6A—C5A—C4A	122.2 (5)
C25A—O7A—Ce1A	139.6 (3)	C6A—C5A—H5A	118.9
C27A—O8A—Ce1A	131.2 (3)	C4A—C5A—H5A	118.9
C18B—O5B—Ce1B	134.3 (3)	C18B—C17B—N3B	126.9 (5)
C9B—N2B—C10B	95.6 (5)	C18B—C17B—S3B	119.2 (5)
C4B—O1B—Ce1B	137.2 (3)	N3B—C17B—S3B	113.8 (4)
C35B—N6B—C34B	108.6 (5)	C23B—C22B—S4B	115.3 (6)
C35B—N6B—C36B	125.3 (5)	C23B—C22B—H22B	122.3
C34B—N6B—C36B	126.1 (5)	S4B—C22B—H22B	122.3
C11B—O3B—Ce1B	140.1 (3)	C22B—C23B—N4B	117.3 (5)
C18A—O5A—Ce1A	141.8 (3)	C22B—C23B—H23B	121.3
C35B—N5B—C29B	109.6 (4)	N4B—C23B—H23B	121.3
C35B—N5B—H5BA	125.2	F1A—C7A—F2AA	102.3 (9)
C29B—N5B—H5BA	125.2	F1A—C7A—F2AB	132.7 (9)
C35A—N6A—C34A	108.5 (5)	F1A—C7A—F3A	94.1 (7)
C35A—N6A—C36A	125.7 (5)	F2AA—C7A—F3A	120.0 (9)
C34A—N6A—C36A	125.8 (5)	F2AB—C7A—F3A	75.1 (10)
C35A—N5A—C29A	109.9 (4)	F1A—C7A—C6A	114.0 (6)
C35A—N5A—H5AA	125.0	F2AA—C7A—C6A	116.0 (6)
C29A—N5A—H5AA	125.0	F2AB—C7A—C6A	113.1 (8)
O4A—C13A—C12A	129.8 (5)	F3A—C7A—C6A	108.2 (7)
O4A—C13A—C14A	112.8 (4)	F8BA—C21B—F9B	95.0 (8)
C12A—C13A—C14A	117.2 (5)	F8BA—C21B—F7B	115.2 (8)
C16B—N3B—C17B	100.9 (6)	F9B—C21B—F7B	104.3 (6)
C2A—N1A—C3A	102.8 (5)	F9B—C21B—F8BB	121.5 (9)
C35B—N7B—H7BA	120.0	F7B—C21B—F8BB	94.5 (8)
C35B—N7B—H7BB	120.0	F8BA—C21B—C20B	118.2 (8)
H7BA—N7B—H7BB	120.0	F9B—C21B—C20B	110.8 (5)
C9A—N2A—C10A	108.0 (6)	F7B—C21B—C20B	111.1 (6)
O2B—C6B—C5B	129.0 (5)	F8BB—C21B—C20B	112.7 (8)
O2B—C6B—C7B	114.0 (4)	C2B—C1B—S1B	111.9 (5)
C5B—C6B—C7B	117.0 (5)	C2B—C1B—H1B	124.1
C33B—C34B—N6B	131.5 (6)	S1B—C1B—H1B	124.1
C33B—C34B—C29B	121.5 (6)	C32B—C31B—C30B	120.9 (7)
N6B—C34B—C29B	107.0 (5)	C32B—C31B—H31B	119.6
C11B—C10B—N2B	124.8 (5)	C30B—C31B—H31B	119.6
C11B—C10B—S2B	118.2 (4)	C16A—C15A—S3A	113.9 (6)
N2B—C10B—S2B	117.0 (3)	C16A—C15A—H15A	123.1
C35A—N7A—H7AA	120.0	S3A—C15A—H15A	123.1
C35A—N7A—H7AB	120.0	C23A—C22A—S4A	112.6 (5)
H7AA—N7A—H7AB	120.0	C23A—C22A—H22A	123.7
N4A—C24A—C25A	129.2 (5)	S4A—C22A—H22A	123.7
N4A—C24A—S4A	111.9 (4)	C15A—C16A—N3A	116.8 (6)
C25A—C24A—S4A	118.9 (4)	C15A—C16A—H16A	121.6
O3A—C11A—C12A	123.2 (5)	N3A—C16A—H16A	121.6
O3A—C11A—C10A	118.0 (5)	C7A—F3A—F2AB	52.3 (8)
C12A—C11A—C10A	118.7 (5)	C34A—C33A—C32A	116.8 (7)
O6A—C20A—C19A	130.0 (5)	C34A—C33A—H33A	121.6
O6A—C20A—C21A	112.3 (5)	C32A—C33A—H33A	121.6
C19A—C20A—C21A	117.6 (5)	F12B—C28B—F1	93.4 (13)
O4B—C13B—C12B	130.2 (5)	F12B—C28B—F10B	100.6 (7)
O4B—C13B—C14B	113.2 (5)	F1—C28B—F10B	120.3 (11)
C12B—C13B—C14B	116.5 (5)	F12B—C28B—F2	125.4 (12)
O3B—C11B—C12B	123.4 (5)	F10B—C28B—F2	89.4 (13)
O3B—C11B—C10B	116.6 (5)	F12B—C28B—C27B	112.1 (8)
C12B—C11B—C10B	119.8 (5)	F1—C28B—C27B	115.0 (7)
C13B—C12B—C11B	123.0 (5)	F10B—C28B—C27B	112.3 (7)
C13B—C12B—H12B	118.5	F2—C28B—C27B	112.9 (8)
C11B—C12B—H12B	118.5	C1B—C2B—N1B	115.2 (5)
O5A—C18A—C19A	122.7 (5)	C1B—C2B—H2B	122.4
O5A—C18A—C17A	116.5 (5)	N1B—C2B—H2B	122.4
C19A—C18A—C17A	120.7 (5)	C29A—C30A—C31A	116.7 (7)
N5B—C35B—N7B	125.1 (5)	C29A—C30A—H30A	121.7
N5B—C35B—N6B	109.0 (5)	C31A—C30A—H30A	121.7
N7B—C35B—N6B	125.9 (5)	C8A—C9A—N2A	116.4 (7)
C30B—C29B—C34B	122.3 (6)	C8A—C9A—H9A	121.8
C30B—C29B—N5B	131.9 (6)	N2A—C9A—H9A	121.8
C34B—C29B—N5B	105.8 (5)	C1A—C2A—N1A	117.1 (5)
F4A—C14A—F6A	105.4 (6)	C1A—C2A—H2A	121.4
F4A—C14A—F5A	107.0 (6)	N1A—C2A—H2A	121.4
F6A—C14A—F5A	106.1 (6)	C9A—C8A—S2A	113.4 (6)
F4A—C14A—C13A	111.0 (5)	C9A—C8A—H8A	123.3
F6A—C14A—C13A	111.5 (6)	S2A—C8A—H8A	123.3
F5A—C14A—C13A	115.2 (5)	C8B—C9B—N2B	120.5 (6)
C13A—C12A—C11A	123.0 (5)	C8B—C9B—H9B	119.7
C13A—C12A—H12A	118.5	N2B—C9B—H9B	119.7
C11A—C12A—H12A	118.5	C31B—C32B—C33B	122.2 (7)
C7A—F2AB—F3A	52.6 (7)	C31B—C32B—H32B	118.9
O7A—C25A—C26A	122.7 (5)	C33B—C32B—H32B	118.9
O7A—C25A—C24A	117.5 (5)	F10A—C28A—F12A	104.3 (8)
C26A—C25A—C24A	119.9 (5)	F10A—C28A—F4	122.1 (9)
O1B—C4B—C5B	123.5 (5)	F12A—C28A—F4	94.0 (10)
O1B—C4B—C3B	118.2 (4)	F10A—C28A—F3	93.2 (9)
C5B—C4B—C3B	118.3 (4)	F12A—C28A—F3	124.0 (10)
N2A—C10A—C11A	129.6 (5)	F10A—C28A—C27A	111.8 (8)
N2A—C10A—S2A	112.0 (4)	F12A—C28A—C27A	109.0 (7)
C11A—C10A—S2A	118.3 (4)	F4—C28A—C27A	112.8 (8)
N3A—C17A—C18A	127.6 (5)	F3—C28A—C27A	112.7 (8)
N3A—C17A—S3A	113.7 (4)	C9B—C8B—S2B	114.6 (6)
C18A—C17A—S3A	118.6 (4)	C9B—C8B—H8B	122.7
O5B—C18B—C19B	123.7 (5)	S2B—C8B—H8B	122.7
O5B—C18B—C17B	117.8 (5)	C15B—C16B—N3B	116.5 (7)
C19B—C18B—C17B	118.5 (5)	C15B—C16B—H16B	121.8
C17A—N3A—C16A	104.7 (6)	N3B—C16B—H16B	121.8
N1B—C3B—C4B	128.9 (5)	C2A—C1A—S1A	113.0 (5)
N1B—C3B—S1B	112.1 (4)	C2A—C1A—H1A	123.5
C4B—C3B—S1B	119.0 (4)	S1A—C1A—H1A	123.5
C30A—C29A—N5A	132.4 (6)	C32A—C31A—C30A	121.8 (8)
C30A—C29A—C34A	121.7 (6)	C32A—C31A—H31A	119.1
N5A—C29A—C34A	105.9 (5)	C30A—C31A—H31A	119.1
C6B—C5B—C4B	124.7 (5)	C22A—C23A—N4A	114.8 (6)
C6B—C5B—H5B	117.6	C22A—C23A—H23A	122.6
C4B—C5B—H5B	117.6	N4A—C23A—H23A	122.6
C33A—C34A—C29A	121.1 (6)	C31A—C32A—C33A	121.9 (7)
C33A—C34A—N6A	131.9 (6)	C31A—C32A—H32A	119.1
C29A—C34A—N6A	106.7 (5)	C33A—C32A—H32A	119.1
F6B—C14B—F4B	104.0 (6)	C16B—C15B—S3B	114.6 (7)
F6B—C14B—F5B	108.8 (6)	C16B—C15B—H15B	122.7
F4B—C14B—F5B	105.5 (6)	S3B—C15B—H15B	122.7
			
Ce1A—O4A—C13A—C12A	24.3 (8)	C29B—C34B—C33B—C32B	0.9 (10)
Ce1A—O4A—C13A—C14A	−159.9 (4)	C4B—C3B—N1B—C2B	−179.5 (6)
Ce1B—O2B—C6B—C5B	−17.1 (9)	S1B—C3B—N1B—C2B	0.7 (7)
Ce1B—O2B—C6B—C7B	162.4 (4)	O6B—C20B—C19B—C18B	−3.1 (10)
C35B—N6B—C34B—C33B	178.6 (6)	C21B—C20B—C19B—C18B	172.0 (6)
C36B—N6B—C34B—C33B	−3.4 (10)	O5B—C18B—C19B—C20B	8.9 (9)
C35B—N6B—C34B—C29B	0.0 (6)	C17B—C18B—C19B—C20B	−168.5 (5)
C36B—N6B—C34B—C29B	178.0 (6)	Ce1A—O2A—C4A—C5A	−4.7 (9)
C9B—N2B—C10B—C11B	176.0 (5)	Ce1A—O2A—C4A—C3A	177.8 (3)
C9B—N2B—C10B—S2B	−0.7 (6)	O2A—C4A—C3A—N1A	175.8 (5)
C8B—S2B—C10B—C11B	−176.1 (5)	C5A—C4A—C3A—N1A	−1.9 (8)
C8B—S2B—C10B—N2B	0.8 (5)	O2A—C4A—C3A—S1A	−4.3 (6)
C22A—S4A—C24A—N4A	−1.0 (5)	C5A—C4A—C3A—S1A	178.0 (4)
C22A—S4A—C24A—C25A	178.9 (5)	C2A—N1A—C3A—C4A	−177.4 (5)
Ce1A—O3A—C11A—C12A	−26.8 (8)	C2A—N1A—C3A—S1A	2.6 (5)
Ce1A—O3A—C11A—C10A	157.0 (4)	C1A—S1A—C3A—C4A	177.5 (5)
Ce1A—O6A—C20A—C19A	6.2 (9)	C1A—S1A—C3A—N1A	−2.6 (4)
Ce1A—O6A—C20A—C21A	−178.3 (4)	C25A—C24A—N4A—C23A	−177.4 (6)
Ce1B—O4B—C13B—C12B	6.8 (8)	S4A—C24A—N4A—C23A	2.4 (7)
Ce1B—O4B—C13B—C14B	−169.0 (4)	O2B—C6B—C7B—F1B	−51.6 (8)
Ce1B—O3B—C11B—C12B	−18.8 (9)	C5B—C6B—C7B—F1B	127.9 (6)
Ce1B—O3B—C11B—C10B	157.4 (4)	O2B—C6B—C7B—F2B	−174.4 (6)
N2B—C10B—C11B—O3B	−177.4 (5)	C5B—C6B—C7B—F2B	5.1 (9)
S2B—C10B—C11B—O3B	−0.8 (7)	O2B—C6B—C7B—F3B	65.4 (7)
N2B—C10B—C11B—C12B	−1.1 (7)	C5B—C6B—C7B—F3B	−115.1 (6)
S2B—C10B—C11B—C12B	175.6 (4)	C29A—N5A—C35A—N7A	176.6 (6)
O4B—C13B—C12B—C11B	−3.7 (9)	C29A—N5A—C35A—N6A	−1.9 (7)
C14B—C13B—C12B—C11B	172.0 (5)	C34A—N6A—C35A—N7A	−176.8 (6)
O3B—C11B—C12B—C13B	8.5 (8)	C36A—N6A—C35A—N7A	2.0 (10)
C10B—C11B—C12B—C13B	−167.6 (5)	C34A—N6A—C35A—N5A	1.7 (7)
Ce1A—O5A—C18A—C19A	3.1 (9)	C36A—N6A—C35A—N5A	−179.5 (6)
Ce1A—O5A—C18A—C17A	−172.9 (4)	O6A—C20A—C21A—F9A	53.5 (7)
C29B—N5B—C35B—N7B	179.8 (5)	C19A—C20A—C21A—F9A	−130.4 (6)
C29B—N5B—C35B—N6B	−0.6 (6)	O6A—C20A—C21A—F7A	−62.9 (7)
C34B—N6B—C35B—N5B	0.4 (6)	C19A—C20A—C21A—F7A	113.2 (6)
C36B—N6B—C35B—N5B	−177.6 (5)	O6A—C20A—C21A—F8A	175.2 (6)
C34B—N6B—C35B—N7B	180.0 (5)	C19A—C20A—C21A—F8A	−8.7 (9)
C36B—N6B—C35B—N7B	2.0 (9)	Ce1A—O1A—C6A—C5A	−19.9 (10)
C33B—C34B—C29B—C30B	−0.5 (9)	Ce1A—O1A—C6A—C7A	156.7 (5)
N6B—C34B—C29B—C30B	178.3 (6)	O7B—C25B—C24B—S4B	−1.4 (7)
C33B—C34B—C29B—N5B	−179.2 (6)	C26B—C25B—C24B—S4B	176.0 (4)
N6B—C34B—C29B—N5B	−0.3 (6)	O7B—C25B—C24B—N4B	175.8 (4)
C35B—N5B—C29B—C30B	−177.9 (6)	C26B—C25B—C24B—N4B	−6.7 (8)
C35B—N5B—C29B—C34B	0.6 (6)	C22B—S4B—C24B—C25B	179.8 (5)
O4A—C13A—C14A—F4A	−66.7 (7)	C22B—S4B—C24B—N4B	2.3 (5)
C12A—C13A—C14A—F4A	109.7 (6)	C23B—N4B—C24B—C25B	−179.0 (5)
O4A—C13A—C14A—F6A	50.5 (7)	C23B—N4B—C24B—S4B	−1.7 (5)
C12A—C13A—C14A—F6A	−133.1 (6)	O1A—C6A—C5A—C4A	5.2 (11)
O4A—C13A—C14A—F5A	171.5 (6)	C7A—C6A—C5A—C4A	−171.3 (6)
C12A—C13A—C14A—F5A	−12.1 (9)	O2A—C4A—C5A—C6A	7.4 (9)
O4A—C13A—C12A—C11A	3.1 (9)	C3A—C4A—C5A—C6A	−175.1 (5)
C14A—C13A—C12A—C11A	−172.5 (5)	O5B—C18B—C17B—N3B	168.5 (5)
O3A—C11A—C12A—C13A	−3.0 (8)	C19B—C18B—C17B—N3B	−14.0 (8)
C10A—C11A—C12A—C13A	173.2 (5)	O5B—C18B—C17B—S3B	−14.4 (7)
Ce1A—O7A—C25A—C26A	−21.0 (9)	C19B—C18B—C17B—S3B	163.1 (5)
Ce1A—O7A—C25A—C24A	158.9 (4)	C16B—N3B—C17B—C18B	177.1 (6)
N4A—C24A—C25A—O7A	−177.4 (6)	C16B—N3B—C17B—S3B	−0.1 (6)
S4A—C24A—C25A—O7A	2.8 (7)	C15B—S3B—C17B—C18B	−178.1 (6)
N4A—C24A—C25A—C26A	2.5 (9)	C15B—S3B—C17B—N3B	−0.7 (6)
S4A—C24A—C25A—C26A	−177.3 (5)	C24B—S4B—C22B—C23B	−2.1 (8)
Ce1B—O1B—C4B—C5B	21.1 (9)	S4B—C22B—C23B—N4B	1.3 (10)
Ce1B—O1B—C4B—C3B	−159.2 (4)	C24B—N4B—C23B—C22B	0.3 (8)
C9A—N2A—C10A—C11A	178.1 (6)	F3A—F2AB—C7A—F1A	81.7 (14)
C9A—N2A—C10A—S2A	0.0 (7)	F3A—F2AB—C7A—C6A	−103.8 (9)
O3A—C11A—C10A—N2A	−164.9 (6)	O1A—C6A—C7A—F1A	−19.6 (10)
C12A—C11A—C10A—N2A	18.8 (9)	C5A—C6A—C7A—F1A	157.5 (8)
O3A—C11A—C10A—S2A	13.1 (7)	O1A—C6A—C7A—F2AA	−138.0 (9)
C12A—C11A—C10A—S2A	−163.3 (4)	C5A—C6A—C7A—F2AA	39.1 (12)
C8A—S2A—C10A—N2A	−1.1 (5)	O1A—C6A—C7A—F2AB	164.8 (12)
C8A—S2A—C10A—C11A	−179.4 (5)	C5A—C6A—C7A—F2AB	−18.1 (14)
O5A—C18A—C17A—N3A	172.3 (5)	O1A—C6A—C7A—F3A	83.7 (8)
C19A—C18A—C17A—N3A	−3.8 (8)	C5A—C6A—C7A—F3A	−99.2 (8)
O5A—C18A—C17A—S3A	−2.8 (7)	O6B—C20B—C21B—F8BA	173.7 (8)
C19A—C18A—C17A—S3A	−179.0 (4)	C19B—C20B—C21B—F8BA	−2.1 (11)
C15A—S3A—C17A—N3A	1.2 (5)	O6B—C20B—C21B—F9B	65.7 (8)
C15A—S3A—C17A—C18A	177.0 (5)	C19B—C20B—C21B—F9B	−110.1 (7)
Ce1B—O5B—C18B—C19B	16.6 (8)	O6B—C20B—C21B—F7B	−49.8 (7)
Ce1B—O5B—C18B—C17B	−166.1 (4)	C19B—C20B—C21B—F7B	134.4 (6)
C18A—C17A—N3A—C16A	−175.8 (6)	O6B—C20B—C21B—F8BB	−154.4 (8)
S3A—C17A—N3A—C16A	−0.5 (6)	C19B—C20B—C21B—F8BB	29.8 (11)
O1B—C4B—C3B—N1B	−177.9 (6)	C3B—S1B—C1B—C2B	−0.7 (6)
C5B—C4B—C3B—N1B	1.9 (9)	C29B—C30B—C31B—C32B	0.5 (11)
O1B—C4B—C3B—S1B	1.9 (7)	C17A—S3A—C15A—C16A	−1.8 (7)
C5B—C4B—C3B—S1B	−178.3 (4)	C24A—S4A—C22A—C23A	−0.9 (7)
C1B—S1B—C3B—N1B	0.0 (5)	S3A—C15A—C16A—N3A	1.9 (10)
C1B—S1B—C3B—C4B	−179.8 (5)	C17A—N3A—C16A—C15A	−0.9 (9)
C35A—N5A—C29A—C30A	−176.2 (7)	F1A—C7A—F3A—F2AB	−133.2 (9)
C35A—N5A—C29A—C34A	1.4 (6)	C6A—C7A—F3A—F2AB	109.9 (9)
O2B—C6B—C5B—C4B	−1.5 (10)	C29A—C34A—C33A—C32A	−0.8 (10)
C7B—C6B—C5B—C4B	179.0 (6)	N6A—C34A—C33A—C32A	−173.9 (7)
O1B—C4B—C5B—C6B	1.0 (10)	O8B—C27B—C28B—F12B	54.9 (10)
C3B—C4B—C5B—C6B	−178.8 (5)	C26B—C27B—C28B—F12B	−125.0 (8)
C30A—C29A—C34A—C33A	2.9 (9)	O8B—C27B—C28B—F1	160.0 (14)
N5A—C29A—C34A—C33A	−175.0 (6)	C26B—C27B—C28B—F1	−19.9 (17)
C30A—C29A—C34A—N6A	177.6 (6)	O8B—C27B—C28B—F10B	−57.6 (10)
N5A—C29A—C34A—N6A	−0.3 (6)	C26B—C27B—C28B—F10B	122.5 (7)
C35A—N6A—C34A—C33A	173.0 (7)	O8B—C27B—C28B—F2	−156.8 (14)
C36A—N6A—C34A—C33A	−5.8 (11)	C26B—C27B—C28B—F2	23.3 (17)
C35A—N6A—C34A—C29A	−0.8 (7)	S1B—C1B—C2B—N1B	1.3 (9)
C36A—N6A—C34A—C29A	−179.6 (6)	C3B—N1B—C2B—C1B	−1.3 (9)
O4B—C13B—C14B—F6B	70.8 (7)	N5A—C29A—C30A—C31A	174.2 (6)
C12B—C13B—C14B—F6B	−105.6 (7)	C34A—C29A—C30A—C31A	−3.1 (10)
O4B—C13B—C14B—F4B	−44.8 (7)	C10A—N2A—C9A—C8A	1.6 (10)
C12B—C13B—C14B—F4B	138.8 (6)	C3A—N1A—C2A—C1A	−1.4 (7)
O4B—C13B—C14B—F5B	−165.9 (6)	N2A—C9A—C8A—S2A	−2.6 (10)
C12B—C13B—C14B—F5B	17.7 (9)	C10A—S2A—C8A—C9A	2.1 (7)
Ce1B—O7B—C25B—C26B	8.8 (8)	C10B—N2B—C9B—C8B	0.3 (10)
Ce1B—O7B—C25B—C24B	−173.9 (3)	C30B—C31B—C32B—C33B	−0.1 (12)
C34B—C29B—C30B—C31B	−0.2 (9)	C34B—C33B—C32B—C31B	−0.6 (11)
N5B—C29B—C30B—C31B	178.1 (6)	O8A—C27A—C28A—F10A	−58.9 (10)
Ce1A—O8A—C27A—C26A	12.4 (11)	C26A—C27A—C28A—F10A	120.4 (8)
Ce1A—O8A—C27A—C28A	−168.5 (5)	O8A—C27A—C28A—F12A	56.0 (10)
O6A—C20A—C19A—C18A	5.0 (9)	C26A—C27A—C28A—F12A	−124.8 (8)
C21A—C20A—C19A—C18A	−170.3 (5)	O8A—C27A—C28A—F4	159.0 (9)
O5A—C18A—C19A—C20A	−9.6 (9)	C26A—C27A—C28A—F4	−21.7 (13)
C17A—C18A—C19A—C20A	166.3 (5)	O8A—C27A—C28A—F3	−162.3 (9)
O8A—C27A—C26A—C25A	−0.3 (12)	C26A—C27A—C28A—F3	17.0 (14)
C28A—C27A—C26A—C25A	−179.5 (7)	N2B—C9B—C8B—S2B	0.1 (12)
O7A—C25A—C26A—C27A	2.8 (10)	C10B—S2B—C8B—C9B	−0.5 (8)
C24A—C25A—C26A—C27A	−177.1 (6)	C17B—N3B—C16B—C15B	1.1 (10)
Ce1B—O6B—C20B—C19B	−26.8 (8)	N1A—C2A—C1A—S1A	−0.3 (9)
Ce1B—O6B—C20B—C21B	158.1 (4)	C3A—S1A—C1A—C2A	1.7 (6)
Ce1B—O8B—C27B—C26B	14.9 (10)	C29A—C30A—C31A—C32A	1.3 (11)
Ce1B—O8B—C27B—C28B	−165.0 (5)	S4A—C22A—C23A—N4A	2.5 (10)
O8B—C27B—C26B—C25B	−3.6 (11)	C24A—N4A—C23A—C22A	−3.2 (10)
C28B—C27B—C26B—C25B	176.3 (7)	C30A—C31A—C32A—C33A	0.7 (13)
O7B—C25B—C26B—C27B	−8.3 (9)	C34A—C33A—C32A—C31A	−1.0 (12)
C24B—C25B—C26B—C27B	174.4 (6)	N3B—C16B—C15B—S3B	−1.7 (12)
N6B—C34B—C33B—C32B	−177.6 (7)	C17B—S3B—C15B—C16B	1.4 (9)

Symmetry code: (i) −*x*+2, −*y*+1, −*z*+1.

### 2-Amino-1-methylbenzimidazolium tetrakis[4,4,4-trifluoro-1-(1,3-thiazol-2-yl)butane-1,3-dionato-κ2O,O']cerium(III) Hydrogen-bond geometry (Å, º)

The rind centroids, some of which are referred to in the text and not the table, are defined as follows: *Cg*2 = S1*A*/C1*A*/C2*A*/N1*A*/C3*A*; *Cg*3 = S2*A*/C8*A*/C9*A*/N2*A*/C10*A*; *Cg*4 = S3*A*/C15*A*/C16*A*/N3*A*/C17*A*; *Cg*6 = S1*B*/C1*B*/C2*B*/N1*B*/C3*B*; *Cg*7 = S2*B*/C8*B*/C9*B*/N2*B*/C10*B*; *Cg*8 = S3*B*/C15*B*/C16*B*/N3*B*/C17*B*; *Cg*9 = S4*B*/C22*B*/C23*B*/N4*B*/C24*B*; *Cg*10 = N5*A*/C29*A*/C34*A*/N6*A*/C35*A*; *Cg*12 = N5*B*/C29*B*/C34*B*/N6*B*/C35*B*; *Cg*13 = C29*B*/C30*B*/C31*B*/C32*B*/C33*B*/C34*B*.

**Table:**

*D*—H···*A*	*D*—H	H···*A*	*D*···*A*	*D*—H···*A*
N5*B*—H5*BA*···O2*B*	0.86	1.98	2.817 (5)	166
N5*A*—H5*AA*···O8*A*	0.86	2.00	2.843 (5)	168
N7*B*—H7*BA*···O4*B*	0.86	2.21	3.055 (6)	169
N7*B*—H7*BB*···F7*B*^ii^	0.86	2.41	3.227 (7)	158
N7*A*—H7*AA*···O6*A*	0.86	2.23	3.051 (6)	160
N7*A*—H7*AB*···F6*A*^iii^	0.86	2.60	3.397 (7)	155
C33*B*—H33*B*···F10*B*^ii^	0.93	2.60	3.495 (9)	161
C1*B*—H1*B*···F6*B*^iii^	0.93	2.46	3.178 (8)	134
C33*A*—H33*A*···F3*A*^iii^	0.93	2.51	3.429 (11)	168
C9*A*—H9*A*···S1*B*^iv^	0.93	3.01	3.941 (8)	174
C2*A*—H2*A*···O6*B*^v^	0.93	2.63	3.433 (7)	145
C8*B*—H8*B*···F8*BB*^vi^	0.93	2.52	3.139 (14)	124
C9*A*—H9*A*···*Cg*6^iv^	0.93	2.85	3.656 (10)	145
C31*A*—H31*A*···*Cg*8^v^	0.93	2.91	3.612 (10)	133
C14*B*—F4*B*···*Cg*7^vii^	1.32 (1)	3.59 (1)	3.906 (8)	94 (1)
C14*B*—F5*B*···*Cg*7^vii^	1.33 (1)	3.51 (1)	3.906 (8)	97 (1)
C21*A*—F8*A*···*Cg*4^i^	1.34 (1)	3.45 (1)	4.119 (7)	111 (1)
*Cg*2···*Cg*10			3.668 (4)	
*Cg*3···*Cg*13^i^			3.772 (5)	
*Cg*9···*Cg*12			3.999 (4)	
*Cg*10···*Cg*2			3.668 (4)	

Symmetry codes: (i) −*x*+2, −*y*+1, −*z*+1; (ii) *x*+1, *y*, *z*; (iii) *x*−1, *y*, *z*; (iv) −*x*+1, −*y*+1, −*z*+1; (v) *x*, *y*−1, *z*; (vi) −*x*, −*y*+1, −*z*; (vii) −*x*+1, −*y*+1, −*z*.
